# Harvestman Phenols and Benzoquinones: Characterisation and Biosynthetic Pathway

**DOI:** 10.3390/molecules180911429

**Published:** 2013-09-16

**Authors:** Daniele F. O. Rocha, Felipe C. Wouters, Dávila S. Zampieri, Timothy J. Brocksom, Glauco Machado, Anita J. Marsaioli

**Affiliations:** 1 Instituto de Química, Universidade Estadual de Campinas, C.P. 6154, 13083-970 Campinas, SP, Brasil; 2 Laboratório de Química Bio-Orgânica, Departamento de Química, Universidade Federal de São Carlos, Caixa Postal 676, 13565-905 São Carlos, SP, Brasil; 3 Departamento de Ecologia, Instituto de Biociências, Universidade de São Paulo, Rua do Matão, trav.14, no. 321, 05508-090 São Paulo, SP, Brasil

**Keywords:** chemical defence, NMR spectroscopy, arthropod biosynthesis, labelled precursors, evolution, chemotaxonomy, Opiliones

## Abstract

Benzoquinones are usually present in arthropod defence exudates. Here, we describe the chemical profiles of 12 harvestman species belonging to the neotropical family Gonyleptidae. Nine of the studied species produced benzoquinones, while three produced alkyl phenols. Two benzoquinones and one phenol exhibited biological activity against bacteria and fungi. We also studied the biosynthesis of 2-ethyl-1,4-benzoquinone by feeding *Magnispina neptunus* individuals with ^13^C-labelled precursors; the benzoquinones were biosynthesised through a polyketide pathway using acetate and propionate building blocks.

## 1. Introduction

Opiliones, which are commonly known as harvestmen or daddy longlegs, compose a large arachnid order with approximately 6,500 species widespread across the World [1]. The large neotropical family Gonyleptidae is chemically and morphologically diverse, comprising nearly 820 species [2]. Gonyleptid scent gland exudates are mainly composed of vinyl ketones and their hetero-Diels-Alder adducts [3,4,5], alkyl phenols [3,6,7,8,9,10] and benzoquinones [3,6,11,12,13,14].

In addition to harvestmen, naturally occurring 1,4-benzoquinones with great structural variety are also found in bacteria, plants and other arthropod orders [15,16]. They are known to be toxic and therefore are employed by beetles [17,18,19,20], earwigs [21], termites [22] and harvestmen [12,13] as a defence against natural predators. Additionally, their antimicrobial activity protects cockchafer larvae [23] and adult harvestmen [11] against pathogens, such as bacteria and fungi. The biosynthesis of 1-hepten-3-one produced by the harvestman *Iporangaia pustulosa* (Gonyleptidae) was recently described [24] as the condensation of one acetate and two propionate units following a polyketide pathway. However, there is no evidence of polyketide synthases (PKS) in harvestmen or in any other arthropod species studied so far [25]. These results claimed that additional evidence of PKS activities lay in the production of other classes of harvestman metabolites, such as benzoquinones.

We describe here the detailed chemical characterisation of the exudates for 12 gonyleptid species, which all contained mixtures of benzoquinones or alkyl phenols. Additionally, the biosynthetic pathway of the 2-ethyl-1,4-benzoquinone produced by *Magnispina neptunus* (Gonyleptidae) was investigatedusing ^13^C-labelled precursors.

## 2. Results and Discussion

### 2.1. Chemical Profile of Gonyleptid Exudates

**Figure 1:**
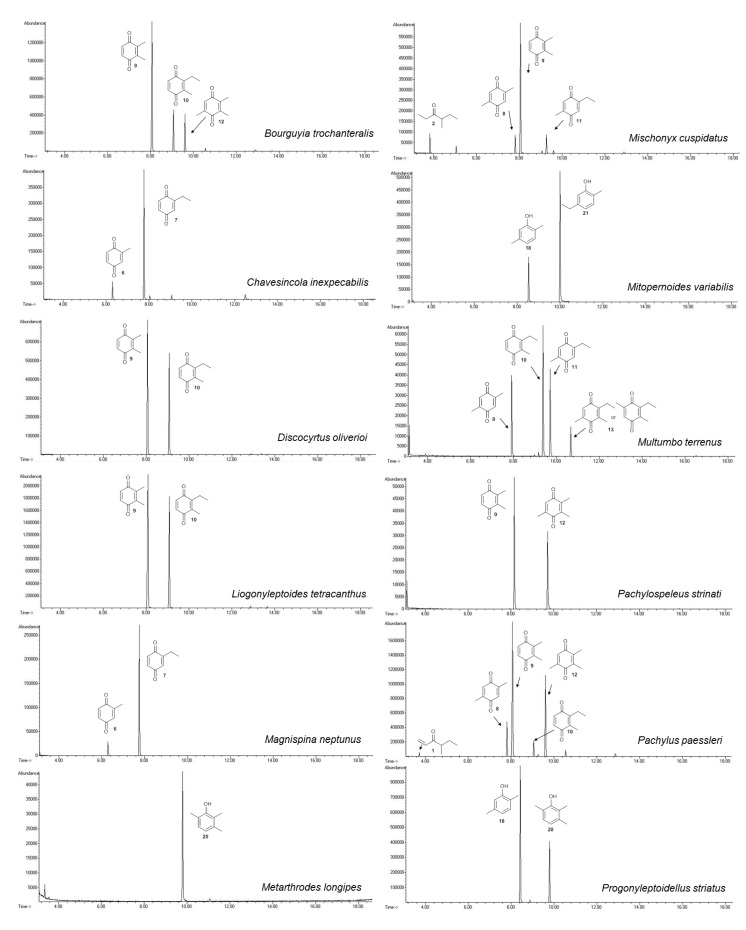
Chromatograms of the harvestman exudates.

The chemical compositions of 12 harvestman species—*Bourguyia trochanteralis* (Bourguyiinae), *Chavesincola inexpectabilis*, *Magnispina neptunus* (both Heteropachylinae), *Discocyrtus oliverioi*, *Pachylus paessleri* (both Pachylinae), *Liogonyleptoides tetracanthus*, *Mischonyx cuspidatus* (both Gonyleptinae), *Metarthrodes longipes* (Caelopyginae), *Mitopernoides variabilis*, *Progonyleptoidellus striatus* (both Progonyleptoidellinae), *Multumbo terrenus* (Hernandariinae) and *Pachylospeleus strinati* (Pachylospeleinae)—were investigated (Table 1). Nine of these species contained mainly mixtures of 1,4-benzoquinones in their scent gland exudate, while the other three produced alkyl phenols in higher abundance (Figure 1). All of the compounds have been characterised by mass spectrometry. Additionally, 2-methylbenzoquinone (**6**), 2-ethylbenzoquinone (**7**), 2,5-dimethylphenol (**18**) and 2,3,6-trimethylphenol (**20**) were fully characterised by ^1^H- and ^13^C-NMR spectroscopy. Co-elutions with synthetic standards were used to confirm the presence of 4-methyl-1-hepten-3-one (**1**) [5], 1-methyl-1,4-benzoquinone (**6**) and 2,5-dimethyl-1,4-benzoquinone (**8**). Additionally, 2,5-dimethyl-phenol (**18**) was compared to a previously characterised natural sample.

#### 2.1.1. Benzoquinones Identification

**Figure 2:**
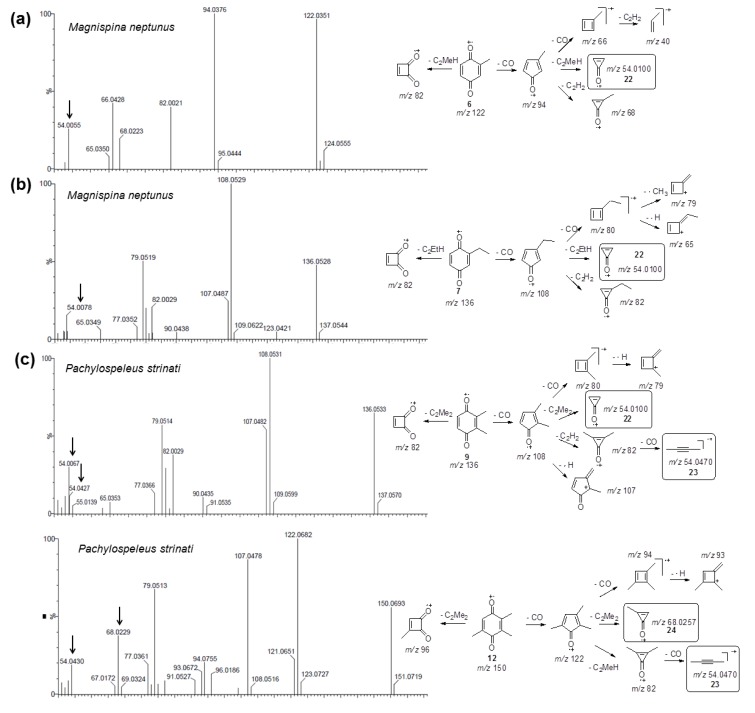
High-resolution mass spectra of benzoquinones from harvestman exudates.

The benzoquinones’ (**6** to **13**) mass spectra have intense molecular ions and characteristic fragmentation patterns, which feature CO and/or C_2_H_2_ loss (Figure 2) [26].

**Table 1:** Compounds detected in defensive secretion of gonyleptid harvestmen, ordered by retention index (RI).

Structure	RI	Characteristic ions [*m/z * (abundance)]	Species	Relative abundance
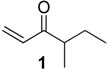	831	112(M^+^,15), 97(12), 84(35), 83(12), 69(12), 58(28), 56(23), 55(100), 41(29)	*Pachylus paessleri*	0.9%
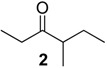	835	114(M^+^,14), 85(10), 57(100), 41(14)	*Mischonyx cuspidatus*	7.0%
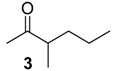	845 *	72(64), 71(19), 70(17), 57(19), 55(14), 43(100), 41(21)	*Mischonyx cuspidatus*	0.2%
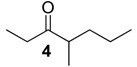	929	128(M^+^, 3), 99(11), 86(60), 71(82), 57(100), 55(13), 43(64), 41(16)	*Mischonyx cuspidatus*	2.8%
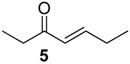	956 *	126(M^+^,26), 111(49), 97(100), 83(21), 69(43), 67(21), 56(26), 55(63), 43(73), 41(78)	*Pachylus paessleri*	0.1%
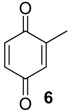	1010	124(45), 123(27), 122(M^+^,100), 94(64), 82(55), 68(31), 66(46), 54(55), 40(24)	*Chavesincola inexpectabilis* *Magnispina neptunus*	10.1% 9.2%
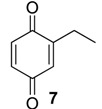	1103	136(M^+^,67), 123(16), 108(100), 107(42), 82(42), 80(18), 79(73), 77(15), 54(52), 53(30)	*Magnispina neptunus* *Chavesincola inexpectabilis*	90.8% 80.2%
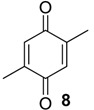	1104	138(14), 137(13), 136(M^+^,100), 108(25), 96(20), 80(19), 79(37), 68(67)	*Multumbo terrenus* *Pachylus paessleri* *Mischonyx cuspidatus*	24.2 %10.3% 8.7%
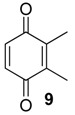	1119	136(M^+^,100), 108(47), 107(47), 82(39), 80(17), 79(41), 54(37), 53(16)	*Mischonyx cuspidatus* *Bourguyia trochanteralis* *Pachylospeleus strinati* *Liogonyleptoides tetracanthus* *Discocyrtus oliverioi* *Pachylus paessleri* *Chavesincola inexpectabilis*	68.3% 65.0% 60.3% 58.6% 57.4% 53.2% 2.4%
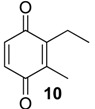	1182	150(M^+^,100), 135(10), 122(31), 121(16), 107(69), 82(20), 79(32), 77(16), 67(10), 54(18), 53(11)	*Discocyrtus oliverioi* *Liogonyleptoides tetracanthus* *Bourguyia trochanteralis* *Pachylus paessleri* *Chavesincola inexpectabilis* *Mischonyx cuspidatus* *Multumbo terrenus*	41.4% 39.9% 17.1% 4.6% 2.7% 1.3% 0.9%
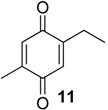	1197	150(M^+^,100), 137(14), 122(45), 121(14), 107(41), 82(13), 79(54), 77(17), 68(24), 54(13), 53(19)	*Multumbo terrenus* *Mischonyx cuspidatus* *Pachylus paessleri*	38.4% 9.3% 0.6%
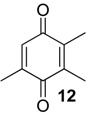	1216	150(M^+^,100), 122(35), 121(19), 107(55), 96(11), 79(39), 77(13), 68(29), 54(16), 53(14)	*Pachylospeleus strinati* *Multumbo terrenus* *Pachylus paessleri* *Bourguyia trochanteralis* *Mischonyx cuspidatus* *Liogonyleptoides tetracanthus*	39.7% 27.6% 27.2% 15.8% 1.5% 0.1%
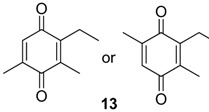	1280	164(M^+^,100), 136(23), 135(13), 121(82), 93(24), 91(15), 77(13), 68(18), 67(13)	*Multumbo terrenus* *Pachylus paessleri* *Bourguyia trochanteralis*	8.9% 2.0% 1.3%
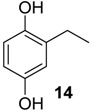	1409 *	138(M^+^,58), 123(100), 107(4), 95(6), 67(10)	*Chavesincola inexpectabilis*	4.6%
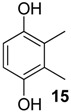	1433 *	138(M^+^,100), 137(29), 123(50), 95(12), 91(13)	*Pachylus paessleri* *Bourguyia trochanteralis* *Liogonyleptoides tetracanthus* *Mischonyx cuspidatus* *Discocyrtus oliverioi*	1.1% 0.8% 0.8% 0.9% 0.5%
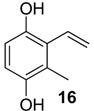	1467 *	150(M^+^,100), 149(21), 121(17), 107(37), 77(15)	*Discocyrtus oliverioi* *Liogonyleptoides tetracanthus*	0.4% 0.1%
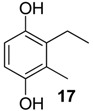	1487 *	152(M^+^,53), 151(11), 138(9), 137(100), 107(8), 79(10), 77(8)	*Liogonyleptoides tetracanthus* *Discocyrtus oliverioi*	0.5% 0.3%
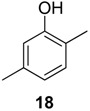	1138	122(M^+^,100), 121(42), 107(96), 91(21), 79(15), 77(27)	*Progonyleptoidellus striatus* *Mitopernoides variabilis*	67.4% 24.5%
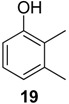	1172 *	122(M^+^,91), 121(32), 107(100), 91(19), 79(16), 77(29)	*Progonyleptoidellus striatus*	1.3%
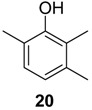	1220	136(M^+^,81), 135(20), 121(100), 91(26), 77(13)	*Metarthrodes longipes* *Progonyleptoidellus striatus*	97.1% 31.3%
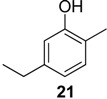	1230	136(M^+^,43), 121(100), 91(15), 77(13)	*Mitopernoides variabilis*	75.5%
(C_4_H_9_)-phenol (**22**)	1315 *	150(M^+^,48), 135(100)	*Metarthrodes longipes*	2.9%

* RI calculated from linear regression from data from the other compounds (see experimental details).

The exudate of *Ma. neptunus* was analysed by CG/TOF-MS; benzoquinones **6** and **7** displayed molecular ions at *m/z* 122 and 136, respectively (Figure 2a,b). The presence of a radical ion at *m/z* 54 (54.0055 for **6** and 54.0078 for **7**) is characteristic of a cyclopropenone radical ion **22** (calculated mass 54.0100), signalling the presence of an asymmetric benzoquinone with substituents on one side of the ring. The benzoquinone present in *Ps. strinati* exudate (Figure 2c) has a molecular ion at *m/z* 136 and could possess either structure **9** or **7**. However, the presence of the two fragments at *m/z* 54.0067 (**22**) and at *m/z* 54.0427 (**23**) (calculated mass 54.0470) indicate that two methyl substituents are on the same side of the benzoquinone, confirming structure **9**. Benzoquinone **12** (Figure 2d), however, has fragments at *m/z* 54.0430 (**23**), indicating the presence of two methyl groups on C-2 and C-3, and at *m/z* 68.0229 (**24**) (calculated mass 68.0257), indicating the presence of a methyl group at C-5. This rationale was utilised to suggest benzoquinone structures **8**, **11** and **13**, which also have methyl substituents at C-5.

**Figure 3:**
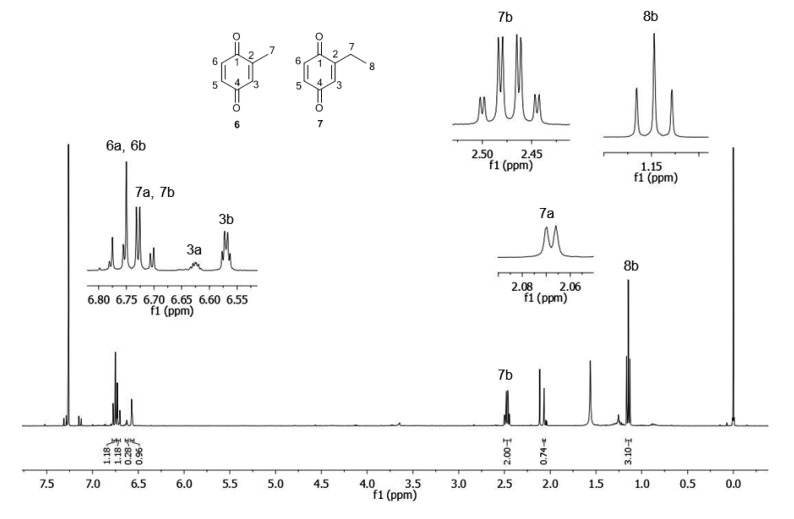
^1^H-NMR spectrum (400.13 MHz, CDCl_3_, TMS) of *Magnispina neptunus* exudate containing benzoquinones **6** and **7** as major components: a-signals refer to benzoquinone **6** and b-signals refer to benzoquinone **7**.

The ^1^H-NMR analyses of *Ma. neptunus* exudate revealed signals consistent with benzoquinones **6** and **7** (Figure 3); the methyl hydrogens at 2.07 ppm corresponded to minor benzoquinone **6** and the triplet at 1.15 ppm, when combined with the quartet at 2.47 ppm, was typical of the ethyl substituent assigned to major benzoquinone **7** [13]. The ^13^C-NMR spectra completed the spectroscopic characterisations of **6** and **7**, except for the assignments of the carbons at the positions C-1/C-4 and C-5/C-6 of 7. Full structural assignment required 2D NMR experiments, such as HSQC and HMBC (Table 2 and Supporting Information). These data were essential for the biosynthetic experiment described below.

**Table 2:** NMR assignments for 2-methyl-1,4-benzoquinone (**6**) and 2-ethyl-1,4-benzo-quinone (**7**) (CDCl_3_, TMS, 400.13 MHz for ^1^H-NMR and 100.61 MHz for ^13^C-NMR).

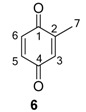			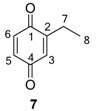	
C	δ_H_	δ_C_ *^a^*	δ_H_	δ_C_ *^b^*
1	-	n.d. *^c^* (C)	-	187.5 (C)
2	-	n.d. *^c^* (C)	-	150.9 (C)
3	6.62 (1H, m)	133.4 (CH)	6.57 (1H, m)	131.7 (CH)
4	-	n.d. *^c^* (C)	-	187.9 (C)
5	6.71 (1H, dd, *^3^J* = 10 Hz; *^4^J* = 2.25 Hz)	136.5 (CH)	6.71 (1H, dd, *^3^J* = 10 Hz; *^4^J* = 2.25 Hz)	136.3 (CH)
6	6.77 (1H, d, *^3^J* = 10 Hz)	136.6 (CH)	6.77 (1H, d, *^3^J* = 10 Hz)	136.8 (CH)
7	2.07 (3H, d, *^4^J* = 1,5 Hz)	15.8 (CH_3_)	2.47 (2H, qd, *^3^J* = 7.5 Hz; *^4^J* = 1.5 Hz)	22.1 (CH_2_)
8	-	-	1.15 (3H, t, *^3^J* = 7.5 Hz)	11.6 (CH_3_)

*^a^* The results from ^13^C-NMR (fully decoupled, DEPT-90 and DEPT-135); *^b^* The results from ^13^C-NMR (fully decoupled, DEPT-90 and DEPT-135), 2D NMR *g*COSY (^1^H-^1^H) and *g*HSQC (^1^H-^13^C ^1^*J*) experiments; *^c^* not detected due to low abundance.

**Figure 4:**
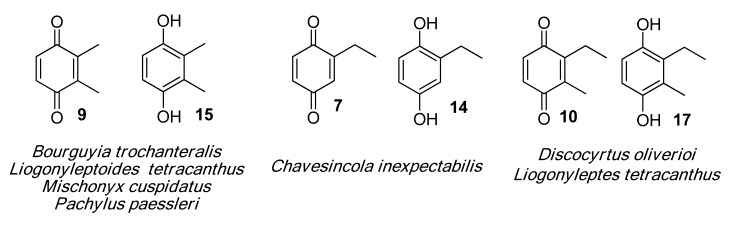
Benzoquinones and corresponding hydroquinones found in gonyleptid exudates.

The 1,4-hydroquinones **14**–**17** were detected in low abundance and identified by mass spectrometry using the peaks at *m/z* 77 and 91, which are typical of alkyl-substituted phenyl derivatives. Additionally, their intense molecular ions possessed two additional mass units when compared with the other corresponding major benzoquinones present in the same exudate (Figure 4). Some 1,4-hydroquinones are also reported for other gonyleptid species alongside their respective benzoquinones [3].

The exudate of *Mi. cuspidatus* has already been studied [3] and the composition was reported to be benzoquinone **9** (46.8%) and its respective hydroquinone **15** (41.5%). In the specimens we studied, the exudate did contain benzoquinone **9**, but only 0.9% of **15** was found. The exudate of *Pa. paessleri* has also been previously studied [14], and the reported composition was similar to ours, except for the presence of the ketone **1** (0.1%) and the hydroquinone **15** (1.1%), reported here for the first time.

#### 2.1.2. Phenols Identification

**Figure 5:**
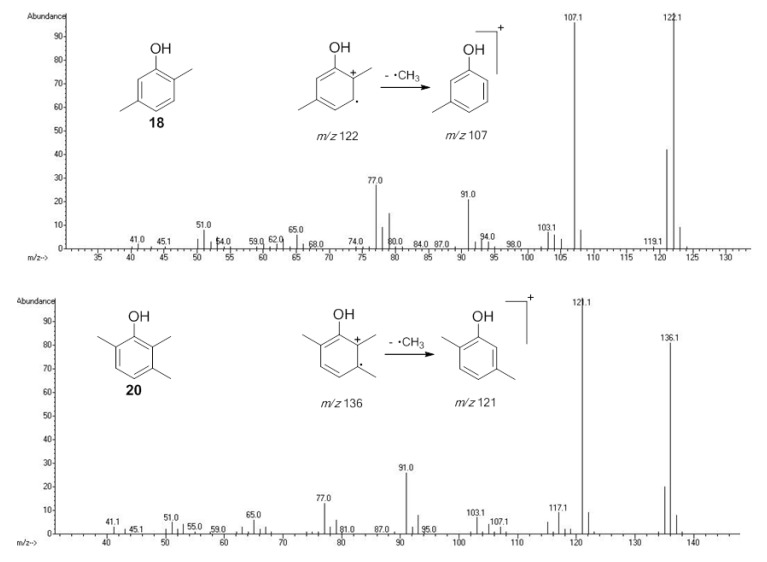
Mass spectra of the alkyl phenols **18** and **20** from *Progonyleptoidellus striatus.*

Phenol derivatives were detected in *Me. longipes*, *Mp. variabilis* and *Pr. striatus*. The exudates of *Pr. striatus* were clean enough to be analysed by mass spectrometry and NMR spectroscopy. The mass fragmentation of **18** and **20** revealed intense molecular ions at *m/z* 122 and 136 and fragments at *m/z* 77 and 91, which are typical of substituted aromatic rings (Figure 5).

**Figure 6:**
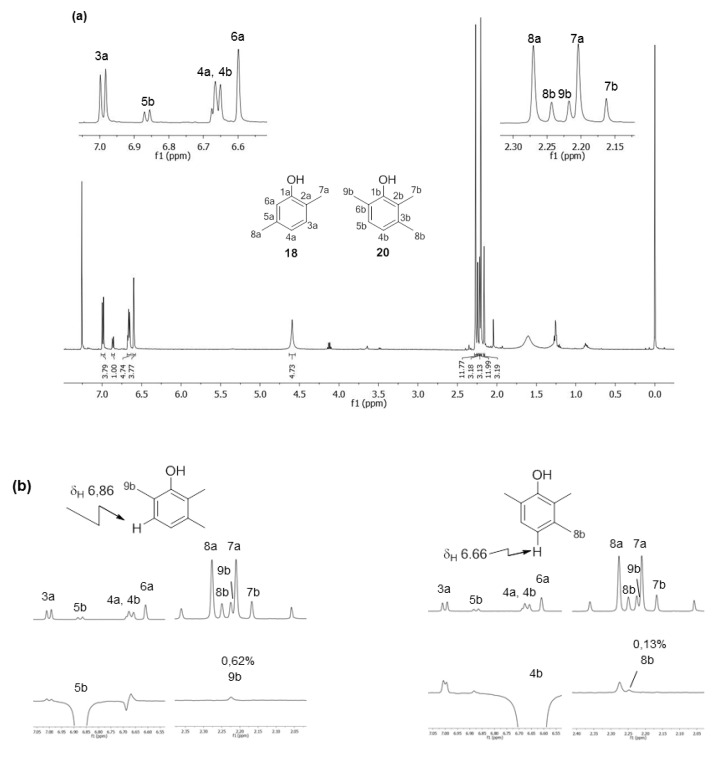
(**a**) ^1^H-NMR spectrum (499.89 MHz, CDCl_3_, TMS) of the harvestman *Progonyleptoidellus striatus* exudate containing phenols **18** and **20** as major components; (**b**) Differential NOE NMR experiments of *Pr. striatus* exudate.

The NMR analysis of *Pr. striatus* revealed the expected methyl substituents and the substitution pattern of **18** (Figure 6a, Table 3). However, the structure of **20** could either be 2,3,6-trimethylphenol or 2,3,4-trimethylphenol. Differential Nuclear Overhauser Effect (NOE) experiments irradiating the aromatic hydrogens at 6.66 and 6.86 ppm enhanced the neighbouring methyl signals 8b (*δ*2.24) and 9b (*δ*2.22), respectively (Figure 6b, Table 3). Therefore, the structure of 2,3,6-trimethylphenol, in which the aromatic hydrogens are spatially next to both methyl groups, was assigned.

**Table 3:** NMR assignments of 2,5-dimethylphenol (**18**) and 2,3,6-trimethylphenol (**20**) (CDCl_3_, TMS, 499.89 MHz for ^1^H-NMR and 125.71 MHz for ^13^C-NMR).

	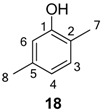		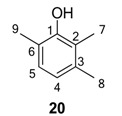	
Х	δ_H_	δ_C_ *^a^*	δ_H_	δ_C_ *^a^*
1	-	153.8 (C)	-	152.1 (C)
2	-	120.6 (C)	-	n.d. *^b^*
3	6.99 (1H, d, *^3^J* = 7.6 Hz)	131.0 (CH)	-	n.d. *^b^*
4	6.66 (1H, d, *^3^J* = 7.6 Hz)	121.6 (CH)	6.66 (1H, d, *^3^J* = 7.6 Hz)	121.9 (CH)
5	-	137.3 (C)	6.86 (1H, d, *^3^J* = 7.6 Hz)	127.6 (CH)
6	6.60 (1H, s)	115.8 (CH)	-	n.d. *^b^*
7	2.20 (3H, s)	15.5 (CH_3_)	2.16 (3H, s)	11.9 (CH_3_)
8	2.27 (3H, s)	21.2 (CH_3_)	2.24 (3H, s)	20.2 (CH_3_)
9	-	-	2.22 (3H, s)	16.1 (CH_3_)

*^a^* The results from ^13^C-NMR (fully decoupled, DEPT-90 and DEPT-135); *^b^* not detected due to the low abundance of this minor compound.

The mass spectrum of 2-methyl-4-phenol (**21**) has an intense fragment at *m/z* 121, corresponding to a methyl loss, which is typical from an ethyl substituent. This identity was confirmed by co-elution with the exudate of the harvestman *Hoplobunus mexicanus* (Stygnopsidae), which was fully characterised by NMR spectroscopy [10].

Within the gonyleptid family, the occurrence of additional phenol derivatives has already been reported for some species, including *Pachyloidellys goliath* [8], *Daguerreia inermis* (both Pachylinae) [3], *Camarana flavipalpi* [9] and *Pseudopachylus longipes* (both Tricommatinae) [3]. Alkyl phenols are also found in species belonging to other families, such as *Cynorta astora* (Cosmetidae), which produces **19** and **21** [6], and *Hoplobunus mexicanus* (Stygnopsidae), which produces **18** and **21** [10].

### 2.2. Antimicrobial Activity of Harvestman Benzoquinones and Phenols

Benzoquinones **6** (from *C. inexpectabilis* and *Ma. neptunus*), **8** (from *Mu. terrenus*, *Pa. paessleri* and *Mi. cuspidatus*) and phenol **18** (from *Ps. striatus* and *Mp. variabilis*) were tested against representative pathogenic microorganisms: a Gram-positive bacterium (*Bacillus pumilus*), a Gram-negative bacterium (*Pseudomonas aeruginosa*) and yeasts (*Candida albicans* and *Rhodotorula glutinis*). The disc diffusion method was applied to obtain the average inhibitory concentrations (data not shown), which were further evaluated in microtiter plates; MIC values are reported in Table 4 (see plates pictured in the Supporting Information).

**Table 4:** Minimal inhibitory concentration values (MIC) for harvestman scent gland components.

Microorganism	MIC (µg/mL)		
	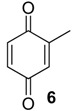	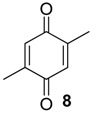	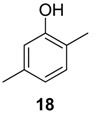
*Bacillus pumilus*	<125	<125	250
*Pseudomonas aeruginosa*	<125	<125	1000
*Candida albicans*	125	<82.5	>500
*Rhodotorula glutinis*	<82.5	<82.5	<82.5

Phenol **18** has lower biological activity than benzoquinones **6** and **8** against all tested microorganisms, except for *R. glutinis*. The biological activities of benzoquinones **6** and **8** were very similar against all tested microorganisms, except for *C. albicans.* The biological activity of the benzoquinones has been assigned to the electrophilic character of the conjugated double bond [27].

Benzoquinone **6** and its corresponding hydroquinone were tested together with other benzoquinone derivatives against *Bacillus subtilis*, *Micrococcus luteus*, and *C. albicans*,showing moderated antimicrobial activity [28]. Ruther and coworkers [23] also found **6** had antimicrobial activity against *Escherichia coli*, *Saccharomyces cerevisiae*, and the entomopathogenic fungi *Metarhizium anisopliae* and *Beauveria brongniartii*.

The antimicrobial activity of phenolic compounds was attributed to membrane permeabilisation, followed by cellular damage [29,30]. A set alkyl phenols were tested against oral bacteria revealing that the ortho-substituents on the phenolic groups decreased the biological activity [31]. In fact, *o*-phenol **18** displayed higher MIC values (Table 4). The dramatic MIC difference displayed by 18 against Gram-positive and Gram-negative bacteria is caused by the lipophilicity of this compound. Therefore, Gram-negative bacteria such as *P. aeruginosa*, have up to 25% of lipidic content and can retain the liposoluble phenols, while Gram-positive species, such as *B. pumilus* have 0% to 3%, therefore allowing the phenol to cause cellular membrane damage [31]. 

### 2.3. Biosynthetic Study of *Magnispina neptunus* Benzoquinone

**Figure 7:**
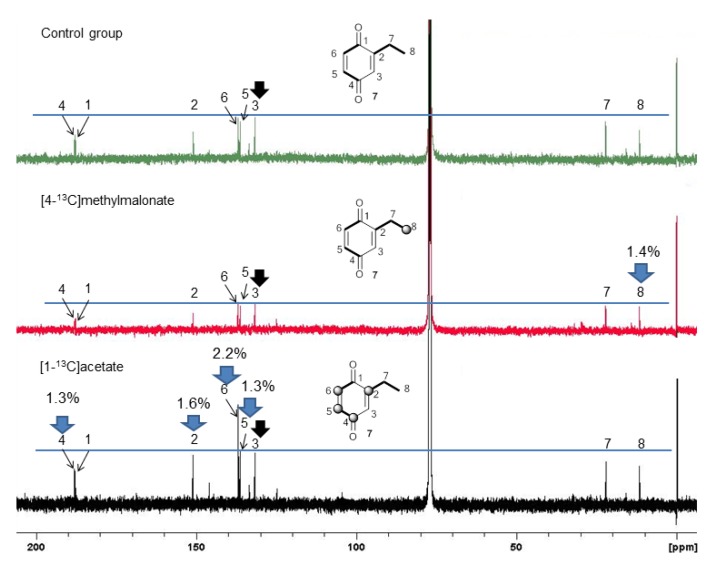
^13^C-NMR spectra (CDCl_3_) of 2-ethyl-1,4-benzoquinone (**7**) after the feeding experiment with *Magnispina neptunus* individuals. Black arrow: control signal; blue arrow: enriched positions. Balls on the structures indicate enriched positions.

**Scheme 1:**
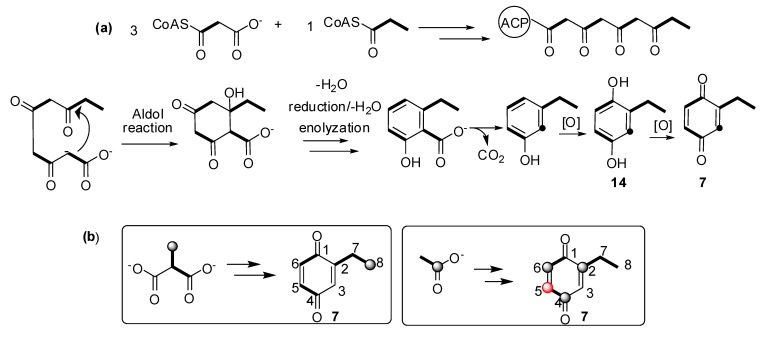
(**a**) Biosynthetic route for **7** in the harvestman *Magnispina neptunus*; (**b**) Labelling pattern of **7** after feeding a labelled precursor to *Ma. neptunus* individuals. Black balls indicate the enriched positions, and the red ball indicates unexpected enrichment.

In a previous report, we described the polyketide pathway for a harvestman vinyl ketone [24], in which the polyketide chain was composed of Pr+Ac+Pr units. To investigate whether this biosynthetic pathway was also present in the quinone producing harvestmen, we selected *Ma. neptunus* as a model species. We added [1-^13^C]acetate and [4-^13^C]methylmalonate as precursors incorporated into the diet of the individuals, and the labelling of 7 was monitored by ^13^C-NMR spectroscopy. The observed enrichment was typical of an aromatic polyketide pathway [32], yielding ^13^C labelling at C-2, C-4, and C-6 of 7, which are alternating carbons on the benzoquinone ring (Figure 7, Scheme 1).

Feeding the individuals [4-^13^C]methylmalonate enriched **7** only at C-8 (Figure 7), which is consistent with the incorporation of a propionyl-CoA starter unit (Scheme 1). This labelling pattern also indicates that the alternative catabolism of propionate to acetate via 3-hydroxypropionate [33] does not occur in *Ma. neptunus* because the positions corresponding to acetate units were not enriched. The same was effect was observed for the vinyl ketone pathway in the harvestman *Iporangaia pustulosa* [24].

[1-^13^C]acetate incorporation enriched C-2, C-4 and C-6 of 7. Positions C-4 and C-6 are clearly labelled due to malonate incorporation. The third extender unit loses its labelled carbon, leaving only the non-labelled carbon at C-3. The unexpected enrichment at C-2 is consistent with the incorporation of a propionate unit and the conversion of [1-^13^C]acetate into [1-^13^C]propionate via succinyl-CoA with a methylmalonyl-CoA mutase, as reported for the harvestman *I. pustulosa* [24]. However, the C_3_ label scrambling observed in *I. pustulosa* was not present in biosynthetic pathway of 7 for *Ma. neptunus*, suggesting a simpler propionate metabolism for this species.

The labelling at C-5 by [1-^13^C] acetate was also unexpected (Scheme 1) because it corresponds to the C-2 of the incorporated malonate unit. The enrichments at C-4 and C-5, which belong to the same acetate unit, are identical (1.3%), excluding the possibility of a measurement error. On the other hand, C-2 and C-6 belong to two C_2_ independent units with 1.6% and 2.2% of enrichment, respectively. Considering that the three C_2_ extender units are malonate, their metabolism should be similar. Therefore, this scrambled labelling suggests that the malonate biosynthesis has more than one route to produce and incorporate this unit onto a hypothetic domain of the PKS. Analogous results were reported for the *I. pustulosa* vinyl ketone biosynthesis [24].

**Scheme 2:**
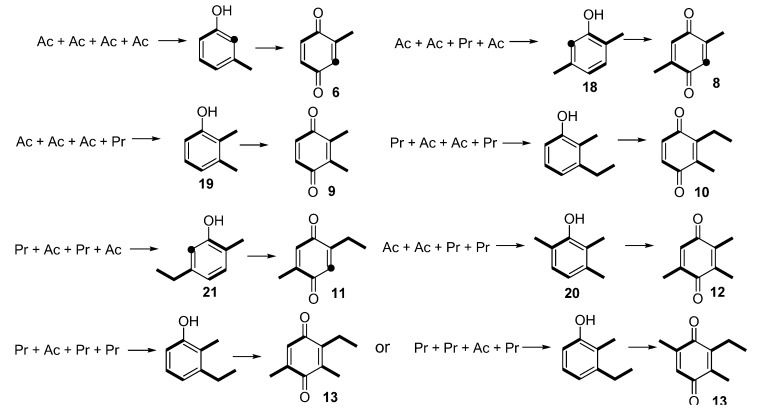
Proposed biosynthetic routes for harvestman benzoquinones and phenols. Black balls indicate a broken acetate unit caused by benzoquinone decarboxylation.

Benzoquinone **6** is the minor constituent of the *Ma. neptunus* exudate and was not detected in the NMR spectra because it had a very low relative abundance. However, its biosynthetic pathway may be similar to **7**, with an acetate starter unit rather than a propionate. The same rationale may be applied to the biosynthesis of the benzoquinones detected in the nine species described in this study, in which changes to the starter and extender units’ assembly provide a different polyketide chain, and therefore generate a set of benzoquinones with different substitution patterns (Scheme 2).

Meinwald and coworkers reported that both aromatic amino acids and carboxylic acids are precursors for unsubstituted 1,4-benzoquinones in the beetle *Eleodes longicollis* [34]. However, the incorporation data for the substituted benzoquinones, such as **6** and **7**, are better accommodated in a polyketide pathway due to the exclusive incorporation of acetate and propionate as starter and extender units, respectively [34]. Sun and Toia studied the biosynthesis of 2,4-dihydroxyacetophenone in the ant *Rhytidoponera chalybaea* revealing the polyketide origin of this aromatic compound in insects [35].

The proposed benzoquinone biosynthetic routes in harvestmen occur via carbonyl enolysation of the cyclised polyketide chain, followed by a decarboxylation (Scheme 1), which is the final step for phenols **18**–**21** from *Me. longipes*, *Mp. variabilis* and *Pr. striatus* (Scheme 2). Eisner and co-workers reported that benzoquinone **9** and phenol **21** from *Zygopachylus albimarginis* (Manaosbiidae) [6] had the same biosynthetic origin for both components. The production of benzoquinones, hydroquinones and phenols by the forked fungus beetle *Bolitotherus cornutus* also suggests that the alkyl benzoquinones and alkyl phenols might share a similar biosynthetic origin [36].

In five of the 12 studied harvestman exudates, the benzoquinone and its corresponding 1,4-hydroquinone occur together (Figure 4). This feature provides additional evidence for the proposed biosynthetic route of phenol *p*-oxidation to provide 1,4-benzoquinones. A classic example of this mechanism is the bombardier beetle (Carabidae), which enzymatically oxidises 1,4-hydroquinones with a catalase producing 1,4-benzoquinones, water and heat [17,18,37]. Beetles of the family Tenebrionidae also produce 1,4-benzoquinones from 1,4-hydroquinone oxidation [15,19,20].

The alkyl and methoxybenzoquinones are also often components of millipede defensive secretions alongside their corresponding hydroquinones [38,39,40,41]. Some millipede species in the families Spirostreptidae and Harpagophoridae secrete benzoquinones **6** and **7**, the hydroquinone **15** and several methoxy substituted benzo- and hydroquinones. According to our hypothesis, the putative biosynthetic route for these compounds is the *p*-oxidation of hydroquinones; however, there have been no labelling experiments using millipedes [39].

Based on these results, as well as on our previous report [24], it can be inferred that harvestmen catabolise propionate to form acetate via succinyl-CoA, followed by the TCA cycle, while insects oxidise the propionate to form acetate via 3-hydroxypropionate [33,42]. The biosynthetic pathway observed in harvestmen most likely relies on the participation of a methylmalonyl-CoA mutase, which is an enzyme exclusive to non-insect arthropods [42,43,44,45]. This feature appears to indicate a key metabolic difference between insects and other arthropods, such as arachnids. The labelling pattern found in the *I. pustulosa* biosynthetic study revealed that this species possesses a complex propionate metabolism, in which the labelling scrambling indicates different loadings of C_3_ starter and extender units [24]. The scrambling was also present in *Ma. neptunus*, but it was observed only for C_2_ extender unit incorporation.

## 3. Experimental

### 3.1. Chemical Profile of Harvestman Exudates

#### 3.1.1. General Methods

The NMR spectra were acquired with either an 11 T Varian Inova instrument, operating at 499.88 MHz for ^1^H-NMR and 125.71 MHz for ^13^C-NMR, or a 5.87 T Bruker Avance DPX, at 250.13 MHz for ^1^H-NMR and 62.89 MHz for ^13^C-NMR. The solvent was CDCl_3_ and tetramethylsylane (TMS) was an internal reference (0.0 ppm). The chemical shifts (*δ*) are reported in ppm and coupling constants *J* are reported in Hz. The GC-MS analyses were performed using an Agilent 6890-5973 system with a DB-5 fused silica capillary column (30 m × 0.25 mm × 0.25 µm). The EIMS were recorded at 70 eV using 3.54 scans·s^−1^ from *m/z* 40 to 400. The oven temperature ranged from 50 to 200 °C at 10 °C·min^−1^ and subsequently to 290 °C at 16 °C·min^−1^. The natural samples were injected in splitless mode, while the synthetic samples were injected in split 1:10 mode. The injector temperature was 250 °C and the detector was maintained at 280 °C; helium was used as the carrier gas. The retention index (RI) [46] was determined using splitless injection mode and temperatures ranging from 50 to 290 °C at a rate of 4 °C·min^−1^ and 7.62 psi; an alkane standard solution C_8_-C_20_ (Fluka) was injected using the same program. The HREIMS were acquired using a Waters GCT premier at 20 scans·s^−1^, at a resolution of 7,000 FWHM, with sub-5 ppm RMS with an internal lock mass correction and electron impact (EI) at 70 eV. The Agilent 7683 operated with oven temperature ranging from 50 to 250 °C at 10 °C·min^−1^ and HP5-MS column with 30 m × 0.25 mm × 0.25 µm for GC analysis. The injection volume was 1 μL in splitless mode. The injector temperature was 270 °C and the detector was kept at 250 °C while using helium as the carrier gas.

#### 3.1.2. Collection of Individuals

The individuals were collected in different places, most of them in the Atlantic Forest in southeastern (SE) Brazil (Table 5) during the wet and warm season (October to March), when the individuals of many gonyleptid species are more active. Individuals of the studied species were taken to the laboratory and kept alive in plastic vials containing a piece of wet cotton to maintain moisture. The scent gland exudates were collected by pressing the gland openings with cotton wool cleaned with bidistilled EtOAc. The liquid absorbed in the cotton wool was washed off with CDCl_3_ (2 mL) for NMR analyses before being eluted with EtOAc (2 mL) for GC-MS analyses. All solvents were of high analytical grade and were doubly distilled before use.

**Table 5:** Identity of the gonyleptid species used in this study. The column “Locality” indicates the places where the individuals were collected in the field and the column “Number of individuals” indicates the sample size used in the chemical analyses.

Species	Locality	Number of individuals
**BOURGOUYINAE**		
*Bourguyia trochanteralis*	Cananéia, São Paulo, SE Brazil	22
**CAELOPYGINAE**		
*Metarthrodes longipes*	Ubatuba, São Paulo, SE Brazil	3
**GONYLEPTINAE**		
*Liogonyleptoides tetracanthus*	Linhares, Espírito Santo, SE Brazil	9
*Mischonyx cuspidatus*	Campinas, São Paulo, SE Brazil	29
**HERNANDARIINAE**		
*Multumbo terrenus*	Teresópolis, Rio de Janeiro, SE Brazil	30
**HETEROPACHYLINAE**		
*Chavesincola inexpectabilis*	Santa Tereza, Espírito Santo, SE Brazil	31
*Magnispina neptunus*	Arraial D’Ajuda, Bahia, NE Brazil	20
**PACHYLINAE**		
*Discocyrtus oliverioi*	Campinas, São Paulo, SE Brazil	11
*Pachylus paessleri*	San Carlos de Apoquindo, Santiago, Chile	24
**PACHYLOSPELEINAE**		
*Pachylospeleus strinati*	Iporanga, São Paulo, SE Brazil	34
**PROGONYLEPTOIDELINAE**		
*Mitopernoides variabilis*	Ubatuba, São Paulo, SE Brazil	9
*Progonyleptoidellus striatus*	Santo André, São Paulo, SE Brazil	10

### 3.2. Antimicrobial Activity

*Bacillus pumillus* (LaBioSin collection) and *Pseudomonas aeruginosa* (CCT 1987) were cultured in Nutrient Broth (NB) (peptone 10 g, glucose 40 g and agar 15 g, and the volume completed to 1 L with distilled water). *Candida albicans* (CCT 0776) and *Rhodotorula glutinis* (CCT 0783) were cultured in yeast-malt extract (YM) Merck (yeast extract 3 g, malt-extract 3 g, peptone 5 g, glucose 10 g and agar 20 g, and the volume completed to 1 L with distilled water). The microorganisms were cultured in 10 mL of the medium for 24 h before the MIC experiment. Aqueous microorganism suspensions (100 µL, 1.5∙× 10^7^ cells·mL^−1^) were added to the wells of a 96 titer plate. The bioactive compounds **6**, **8** and **18** (100 µL) in final concentrations of 1,000, 500, 250 and 125 µg·mL^−1^ for bacteria and 500, 250, 125 and 82.5 µg·mL^−1^ for yeast, diluted in H_2_O/DMSO 95:5 (*v*/*v*) were added in the wells. Positive controls were prepared by substituting the test compounds by either chloramphenicol (4 mg·mL^−1^) for bacteria and ciclopiroxolamine (10 mg·mL^−1^) for the yeast. Negative controls were prepared using only the aqueous DMSO plus inoculum. The plates were incubated at 30 °C for 24 h. Aliquots of 20 µL of aqueous MTT (1 mg·mL^−1^) were added to the wells, and the reduction of the terazolium salt (yellow) to formazan (blue) by living cells was observed within 1 h. All of the tests were run in triplicates.

### 3.3. Biosynthetic Study of Magnispina neptunus

The individuals used in the biosynthetic study were collected at Arraial D’Ajuda, state of Bahia, northeastern Brazil. Before beginning the experiment, a dorso-ventral pressure was applied to all of the individuals to empty their gland sacs. The individuals were divided into two groups and fed with canned dog food containing 5% *w*/*w* of the labelled precursors: [1-^13^C]sodium acetate (Cambridge Isotope Laboratories, CIL, Tewksbury, MA, USA) (n = 40 individuals) and [4-^13^C]sodium methylmalonate (for synthetic procedure see [17]) (n = 33 individuals). The control group was the exudate extracted before initiating each experiment (n = 68 individuals). The experiment was set up over a period of 60 days, and the food was renewed every 48 h. The exudates were collected with dewaxed cotton wool and extracted from the cotton wool with CDCl_3_. ^13^C-NMR spectra of **7** were acquired with a Bruker Avance III 11 tesla operating at 125.75 MHz, 25 °C, acquisition time 1.1 s, 30° pulse, and approximately 40,000 scans, using equal scan numbers for samples within the same experiment (sample and control) [47,48].

## 4. Conclusions

**Scheme 3:**
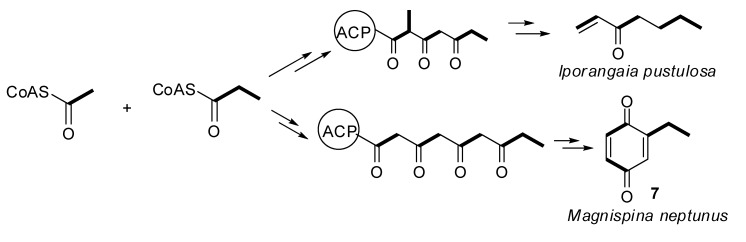
Biosynthetic pathway for vinyl ketone and benzoquinone in harvestmen.

The chemical characterisation of the 12 harvestman exudates provides important information related to the chemotaxonomy of the gonyleptid harvestmen. For three species studied here (*L. tetracanthus*, *Me. longipes* and *Mp. variabilis*), there was no previous chemical characterisation in the literature. The data for these three species, which were not included in the recent phylogeny of the Gonyleptidae, support the notion that the production of benzoquinones is plesiomorphic in the family [49]. Additionally, the production of alkyl-phenols evolved several times independently from the ancestral states of production of benzoquinones and vinyl ketones [49]. These frequent evolutionary transitions agree with the proposed biosynthetic route for benzoquinones, phenols and ketones, all of which begin from acetate and propionate units in a common polyketide pathway. Specifically, our studies with *I. pustulosa* [24] and *Ma. neptunus* indicate that these phylogenetically distant species partially share the biosynthetic pathway for vinyl ketones and benzoquinones, respectively (Scheme 3). Additionally, the scrambled labelling at specific positions of the polyketide chain also indicates that the biosynthetic routes are complex, offering several opportunities for the diversification of the molecules produced within both species.

Most species that produce benzoquinones live on the ground, taking shelter under rocks or rotten logs [49]. In these types of habitats, individuals are likely to be in direct contact with many pathogenic microorganisms [50]. However, most species living on low and high vegetation produce phenols and ketones as the main constituents of their defensive exudates [24]. The results from our experiments concerning antimicrobial activity revealed that the minimal inhibitory concentration values for benzoquinones are consistently lower than for phenols, suggesting that the benzoquinones are more effective at deterring microorganisms. Therefore, the diversification of the chemical compounds in the defensive exudates of gonyleptid harvestmen may be at least partially explained by the differences in habitat-related uses among the species of different subfamilies.

## Supplementary Materials

Supplementary materials can be accessed at: http://www.mdpi.com/1420-3049/18/9/11429/s1.
