# Supplementary material for: Harvestman Phenols and Benzoquinones: Characterisation and Biosynthetic Pathway
Source: Molecules. 2013 Sep 16;18(9):11429–51. doi: 10.3390/molecules180911429 (PMC6270637; doi:10.3390/molecules180911429)

Supplementary Materials

Figure S1. Mass spectra of 1-hexen-3-one (**1**).

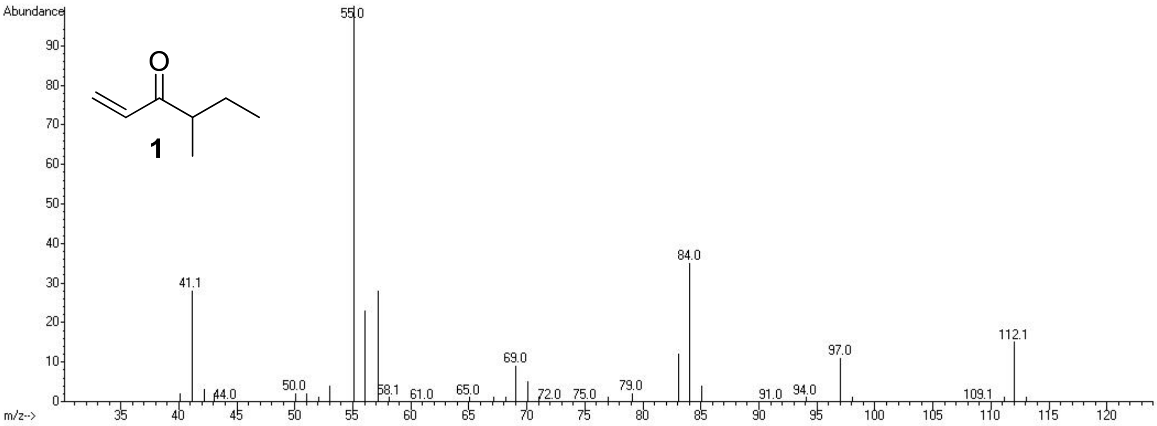

Figure S2. Mass spectra of 3-hexanone (**2**).

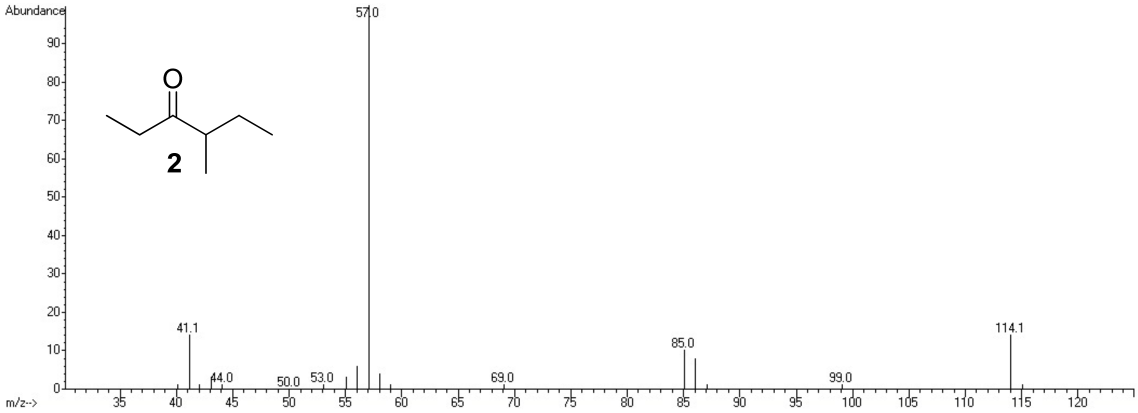

Figure S3. Mass spectra of 3-methyl-2-hexanone (**3**).

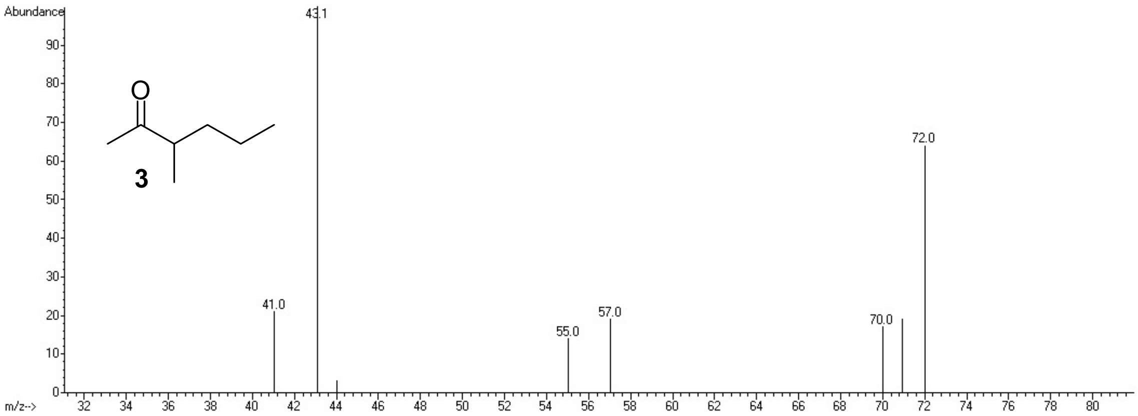

Figure S4. Mass spectra of 3-methyl-3-heptanone (**4**).

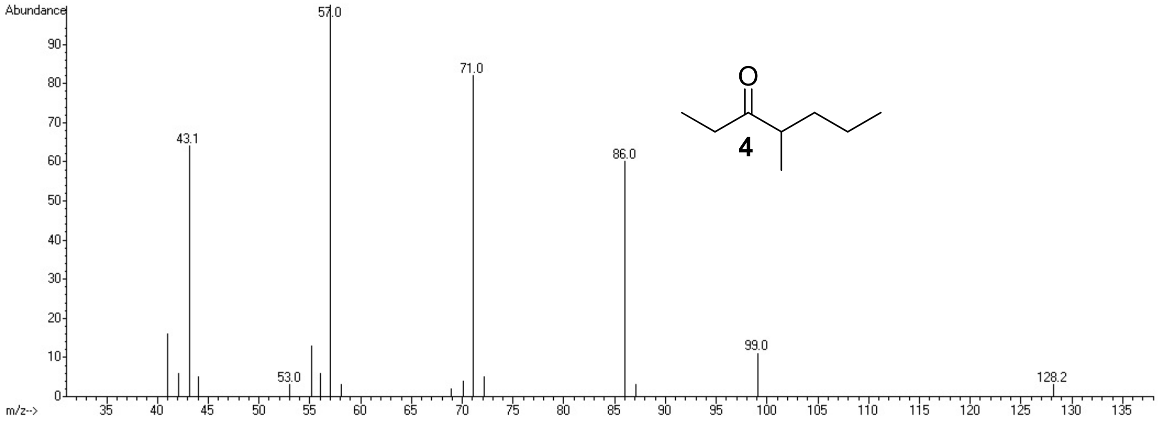

Figure S5. Mass spectra of 4-hepten-3-one (**5**).

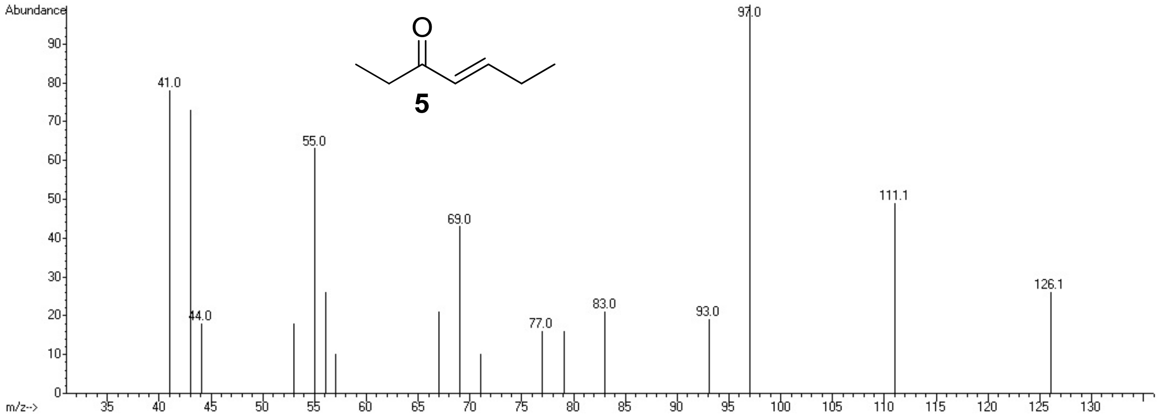

Figure S6. Mass spectra of 2,5-dimethyl-1,4-benzoquinone (**8**).

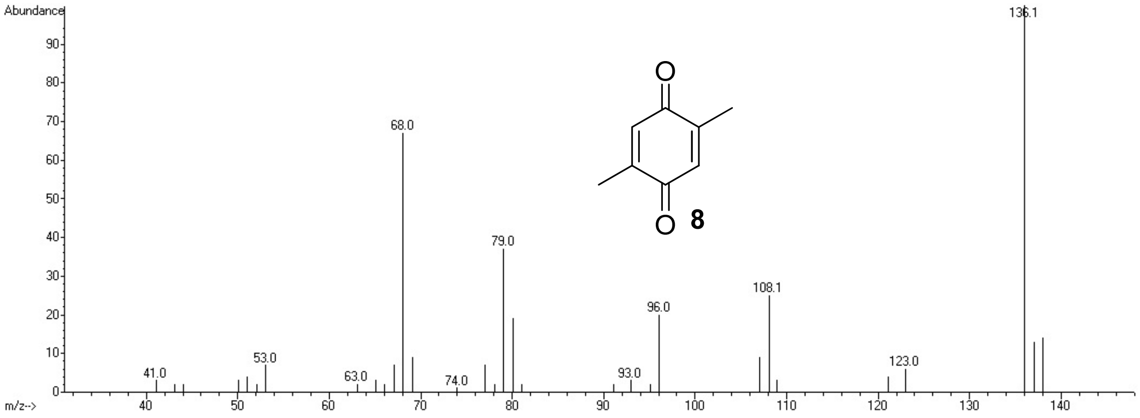

Figure S7. Mass spectra of 2-ethyl,3-methyl-1,4-benzoquinone (**10**).

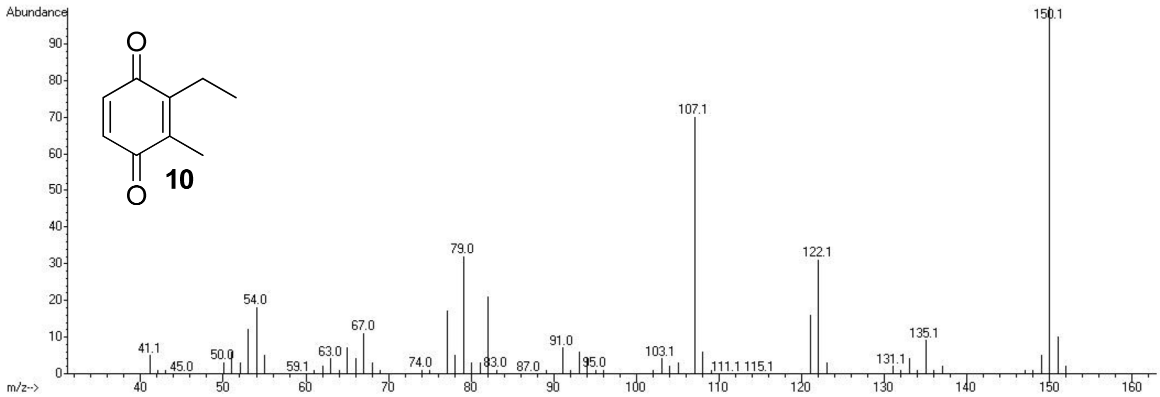

Figure S8. Mass spectra of 2-ethyl,5-methyl-1,4-benzoquinone (**11**).

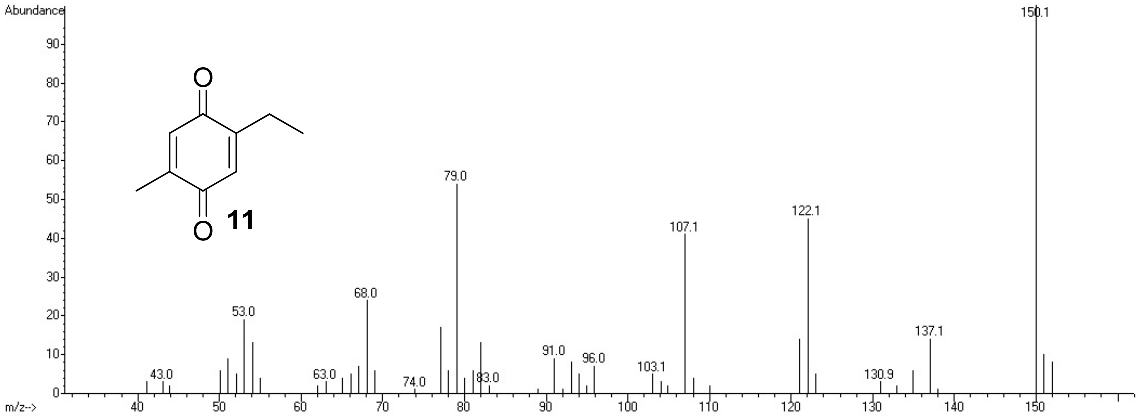

Figure S9. Fragmentation pattern and mass spectra of benzoquinone (**13**).

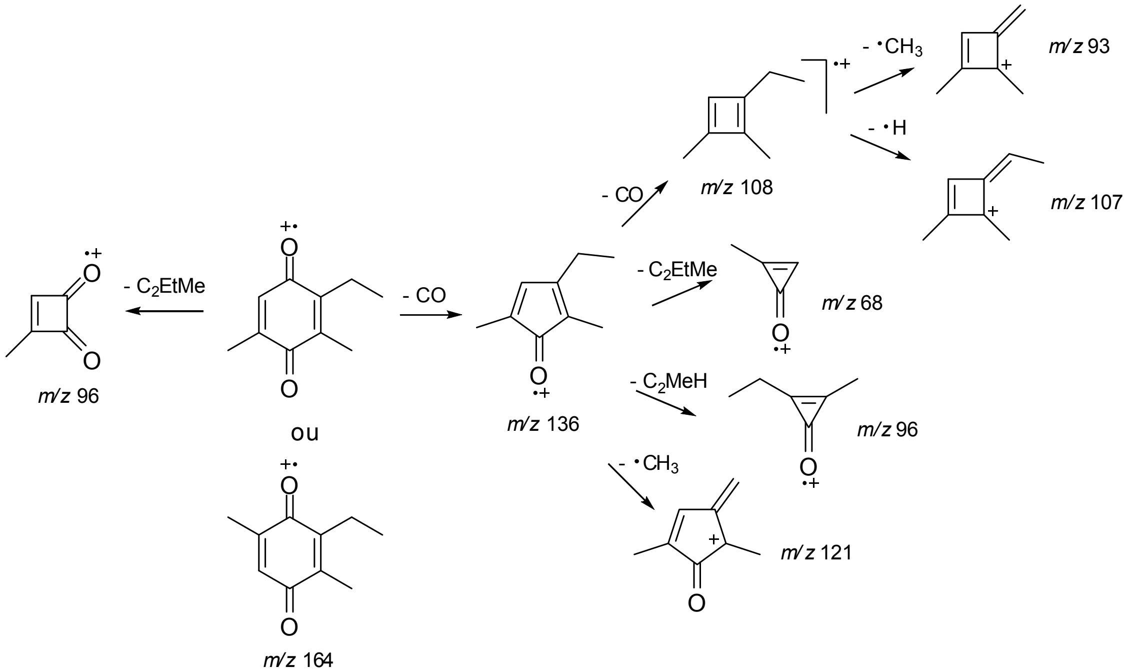

Figure S9. *Cont.*

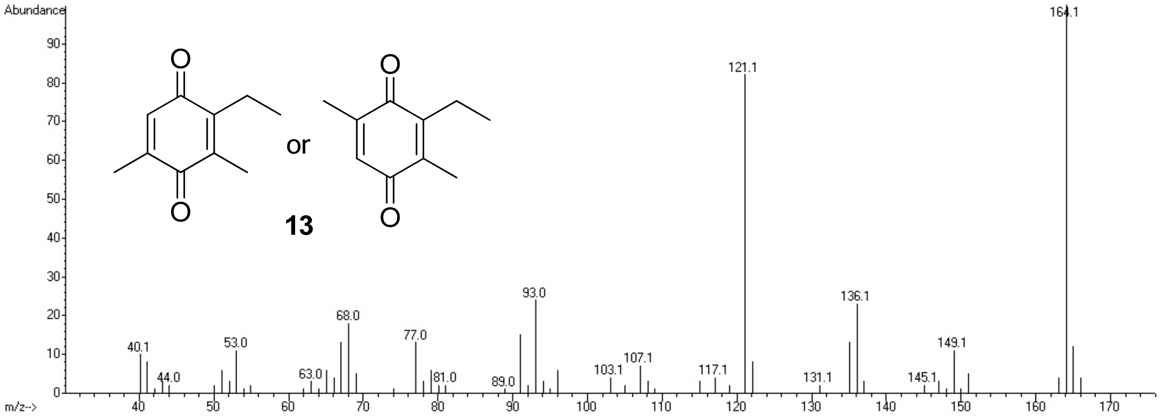

Figure S10. Mass spectra of 2-ethyl-1,4-hydroquinone (**14**).

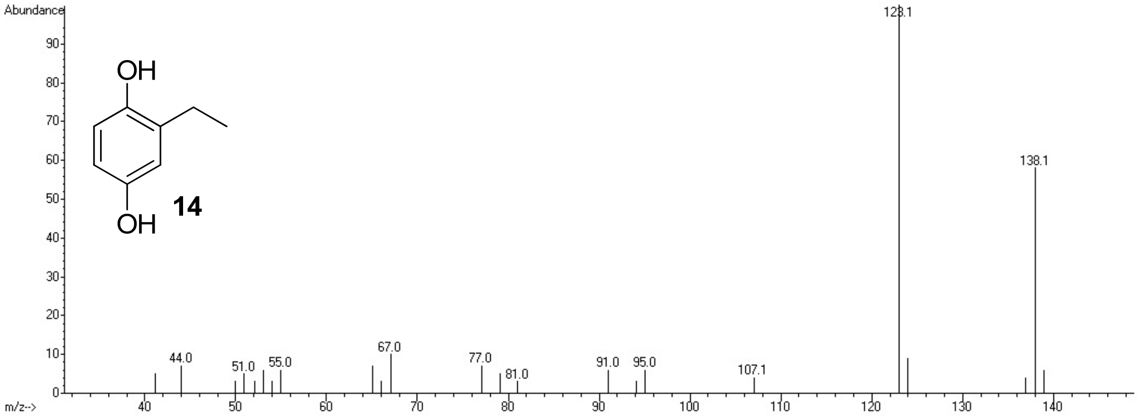

Figure S11. Mass spectra of 2,3-dimethyl,1,4-hydroquinone (**15**).

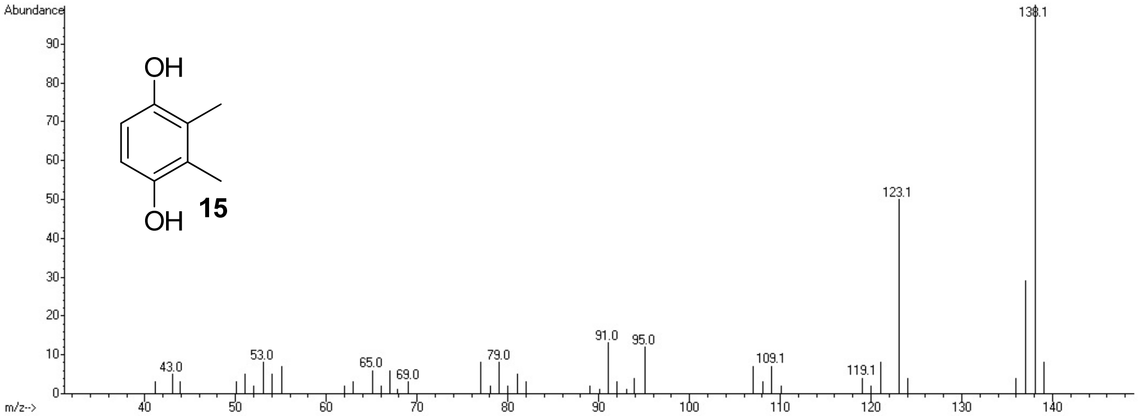

Figure S12. Mass spectra of 2-ethenyl-3-mehyl-1,4-hydroquinone (**16**).

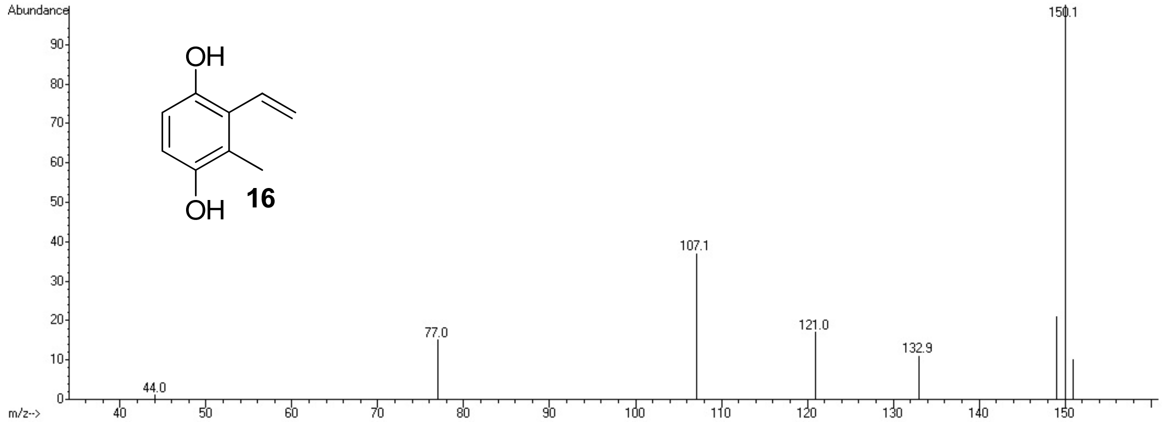

Figure S13. Mass spectra of 2-ethyl-3-mehyl-1,4-hydroquinone (**17**).

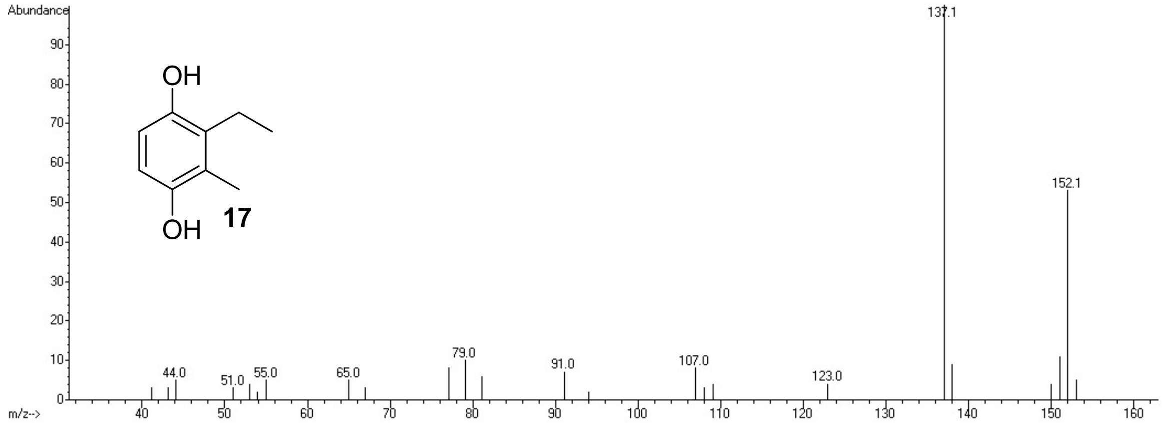

Figure S14. Mass spectra of 2,3-dimethyl-phenol (**19**).

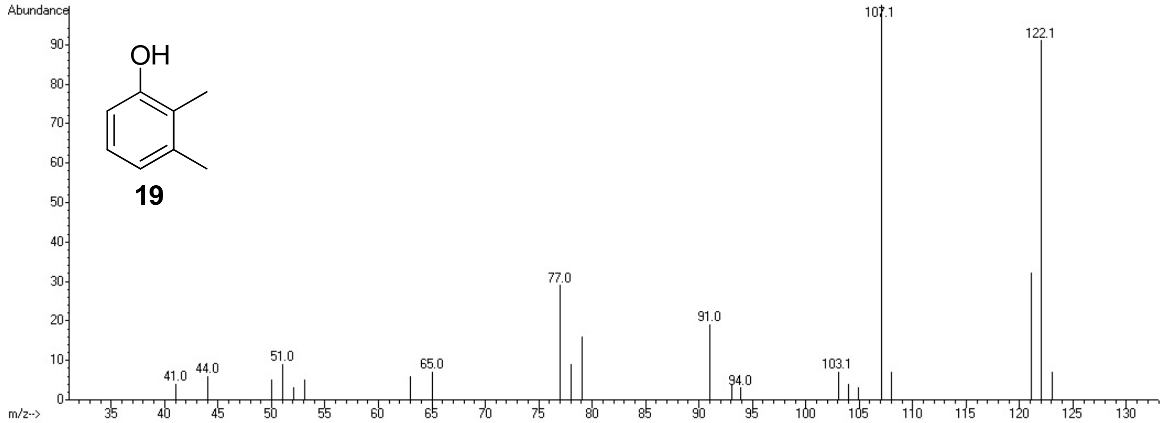

Figure S15. Mass spectra of 2-methyl,5-ethyl-phenol (**21**).

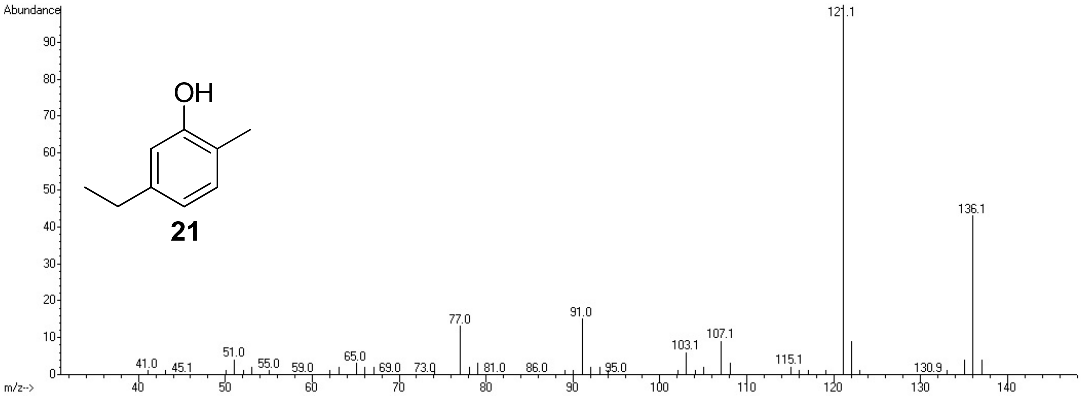

Figure S16. Mass spectra of compound **22**.

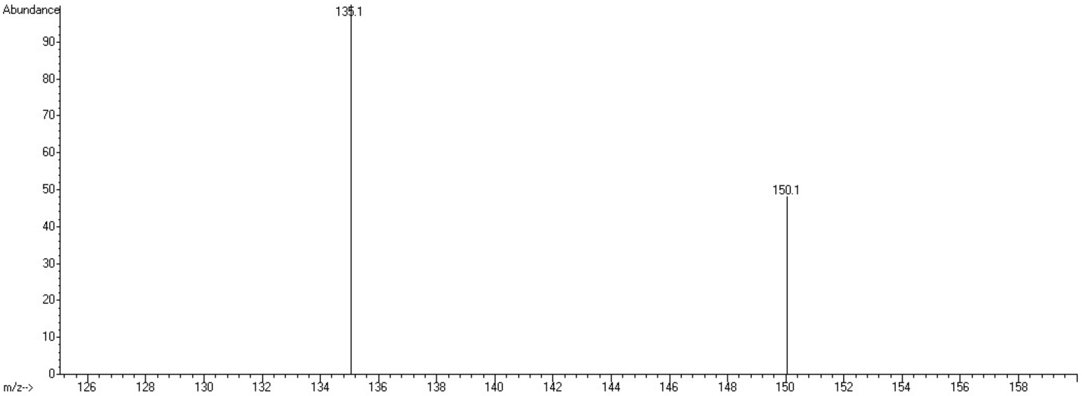

Figure S17. ^{13}C-NMR spectra (100.61 MHz, $CDCl_3$, TMS) of *Magnispina neptunus* exudate containing compounds **6** and **7**.

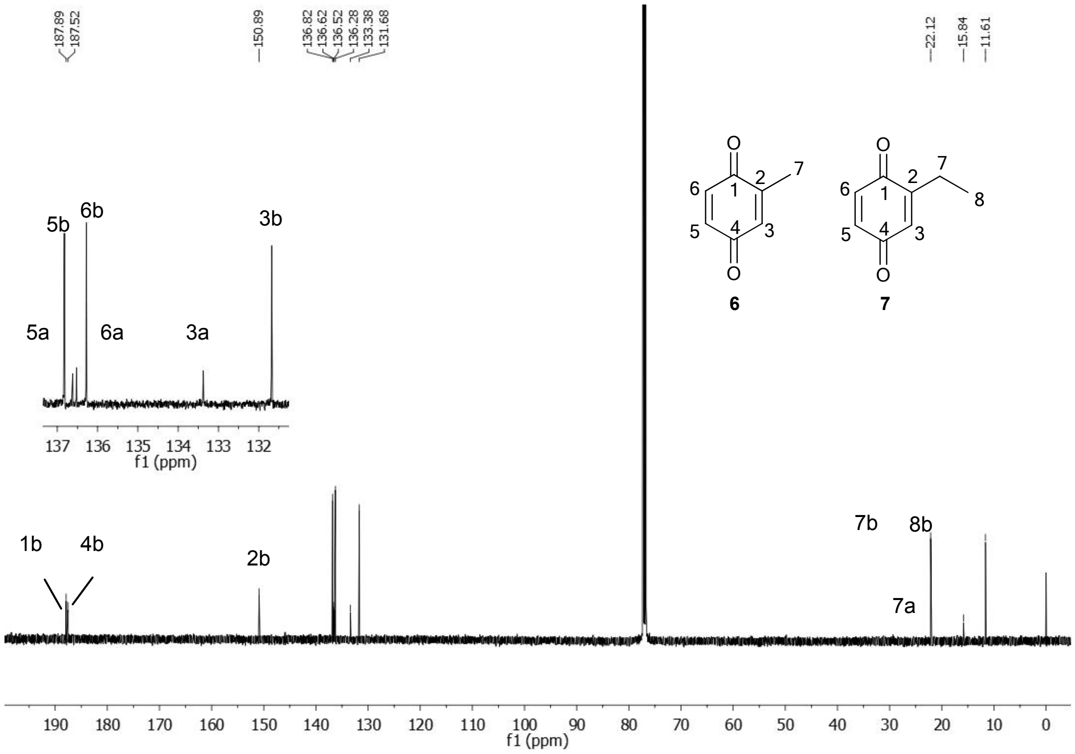

Figure S18. Contour map of 2D NMR ^{1}H, ^{1}H (COSY) (400.13 MHz, $CDCl_3$, TMS) of *Magnispina neptunus* exudate containing compounds **6** and **7**.

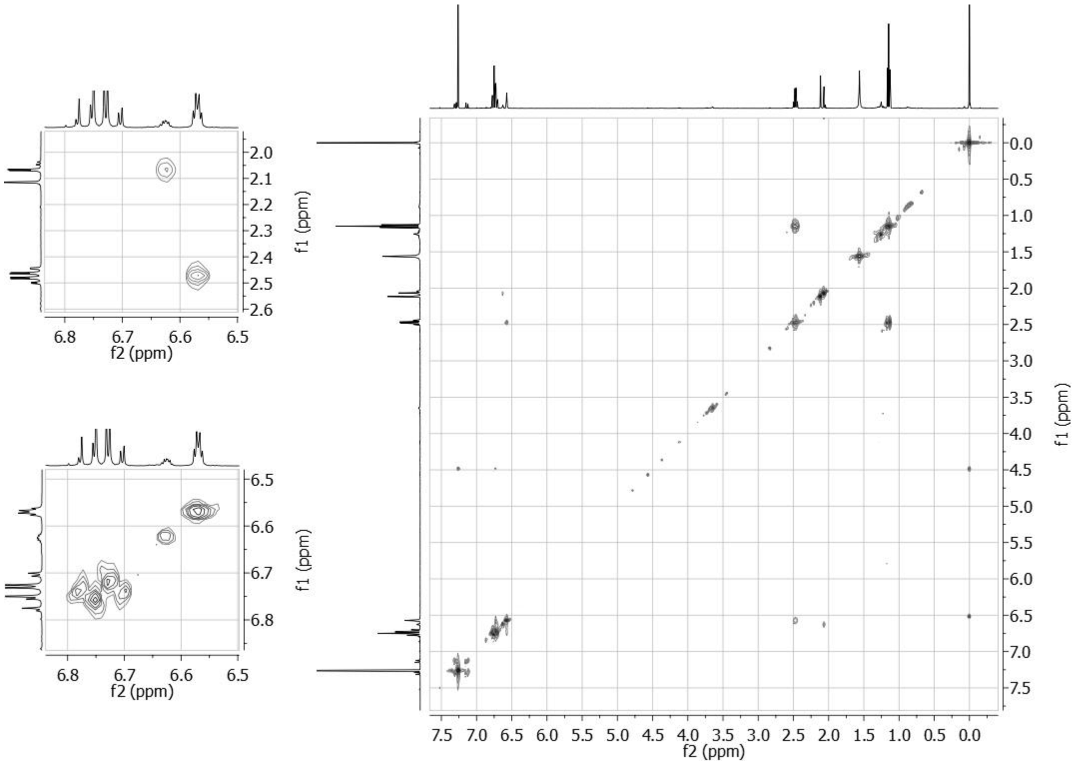

Figure S19. Contour map of 2D NMR ^{1}H (400.13 MHz) and ^{13}C (100.61 MHz) ^{1}J HSQC of *Magnispina neptunus* exudate containing compounds **6** and **7**.

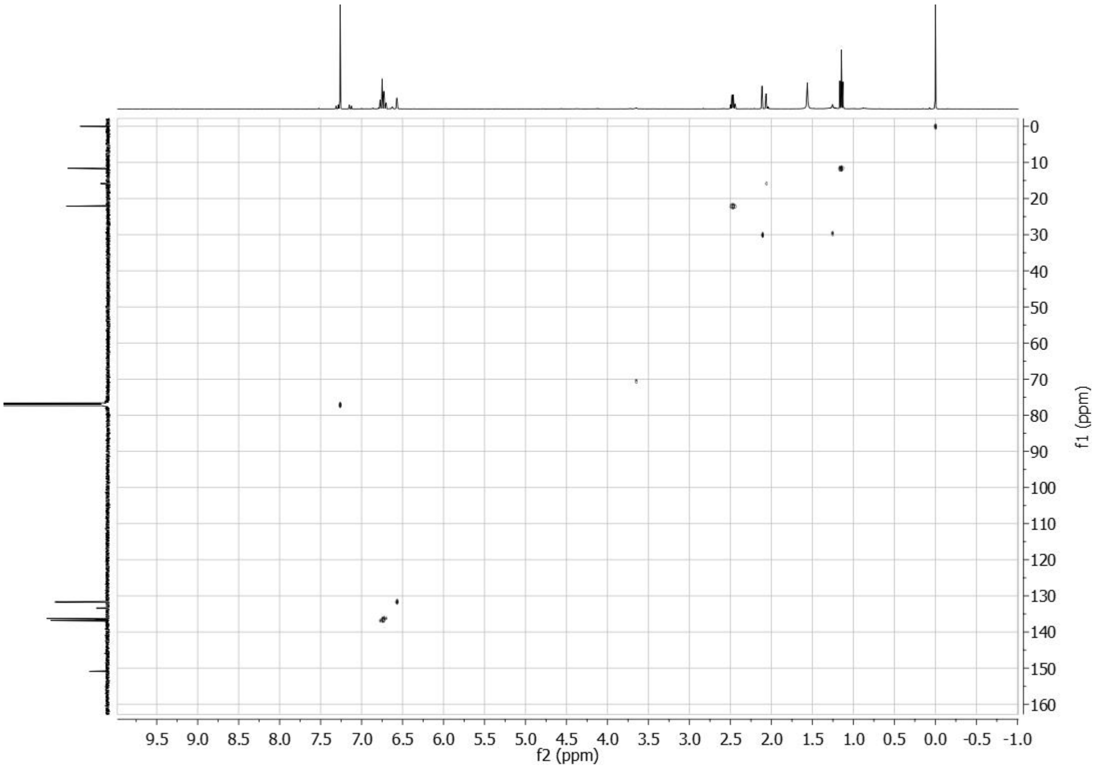

Figure S20. Expansion of contour map of 2D NMR ^{1}H (400.13 MHz) and ^{13}C (100.61 MHz) ^{1}J HSQC of *Magnispina neptunus* exudate containing compounds **6** and **7**.

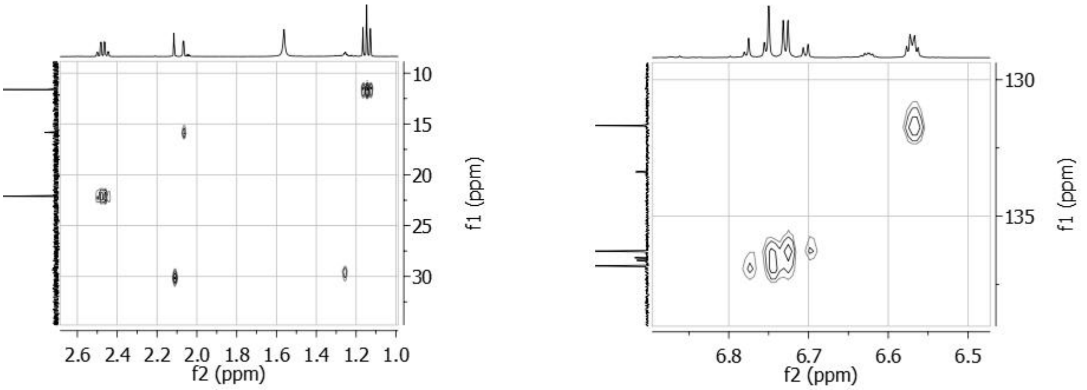

Figure S21. ^{13}C NMR (125.71 MHz, $CDCl_3$, TMS) of *Progonyleptoidellus striatus* exudate containing compounds **18** and **20**.

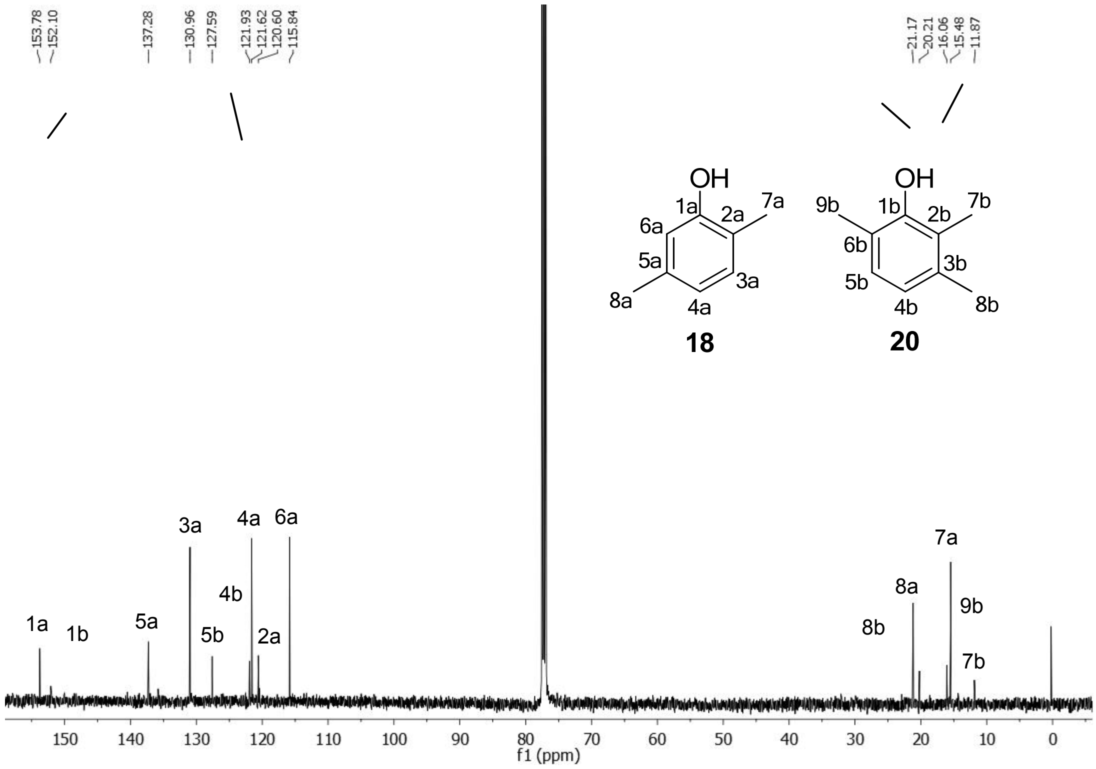

Figure S22. ^{13}C-NMR DEPT 135° e DEPT 90° (125.71 MHz, $CDCl_3$, TMS) of *Progonyleptoidellus striatus* exudate containing compounds **18** and **20**.

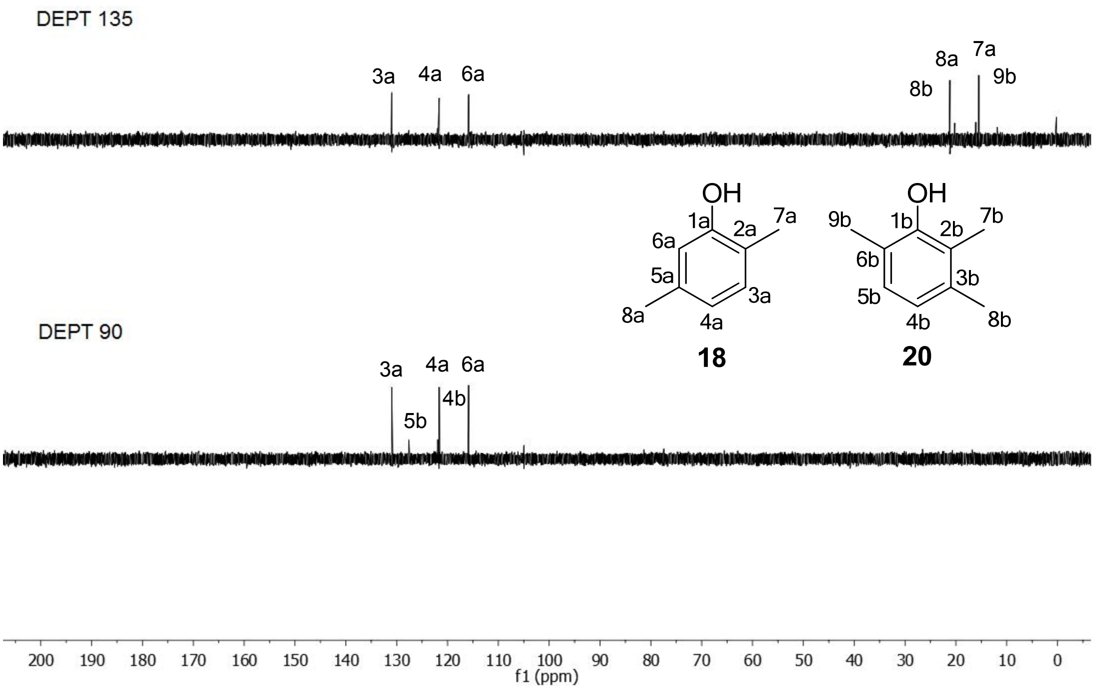

Figure S23. Contour map of 2D NMR ^{1}H, ^{1}H (COSY) (499.89 MHz, $CDCl_3$, TMS) of *Progonyleptoidellus striatus* exudate containing compounds **18** and **20**.

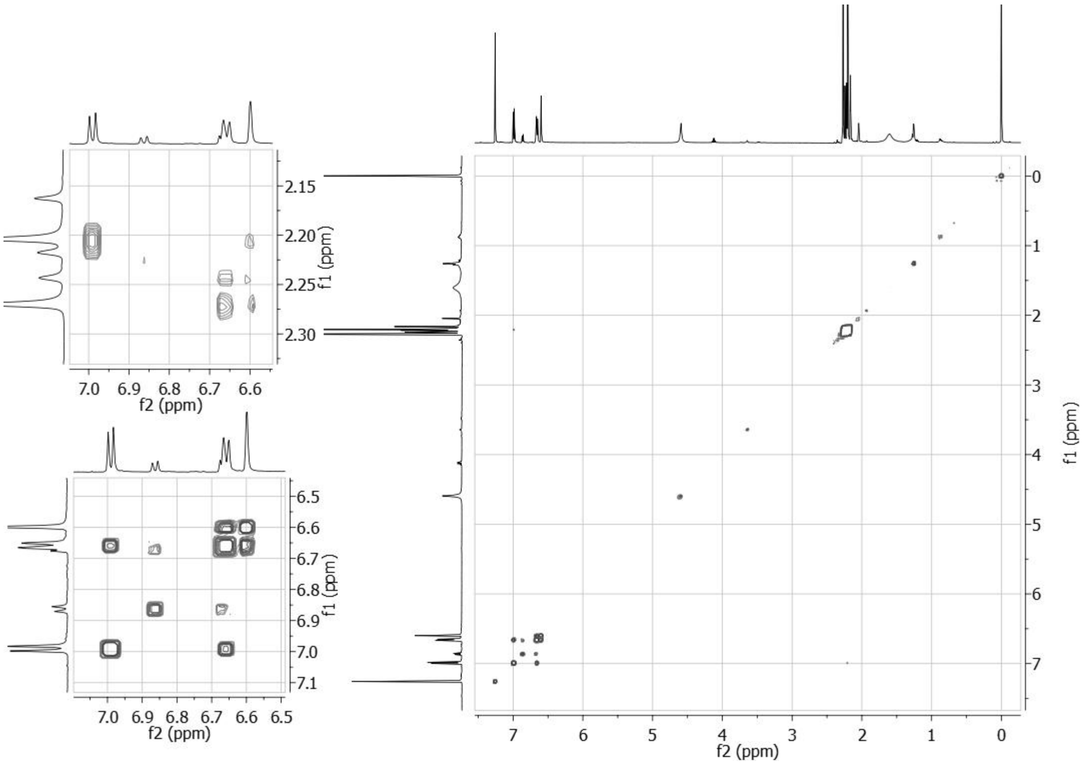

Figure S24. Contour map of 2D NMR ^{1}H (499.89 MHz) and ^{13}C (125.71 MHz) *[1]J* HSQC experiment of *Progonyleptoidellus striatus* exudate containing compounds **18** and **20**.

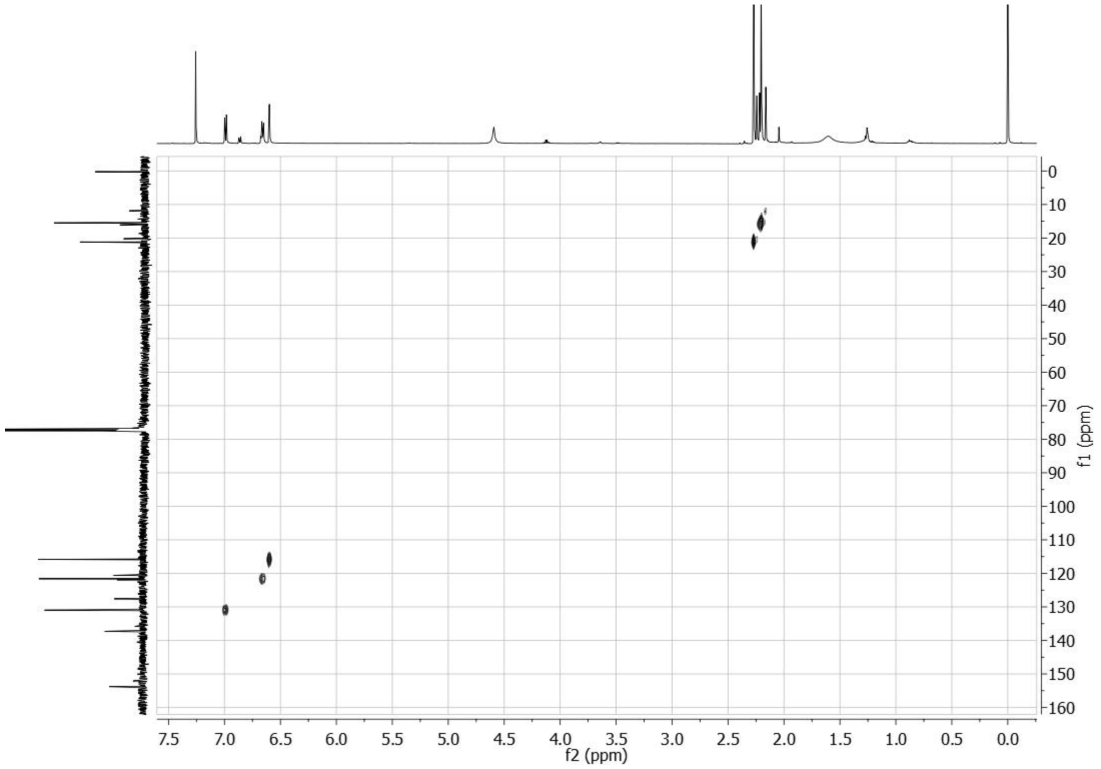

Figure S25. Expansion of contour map of 2D NMR ^{1}H (499.89 MHz) and ^{13}C (125.71 MHz) *[1]J* HSQC experiment of *Progonyleptoidellus striatus* exudate containing compounds **18** and **20**.

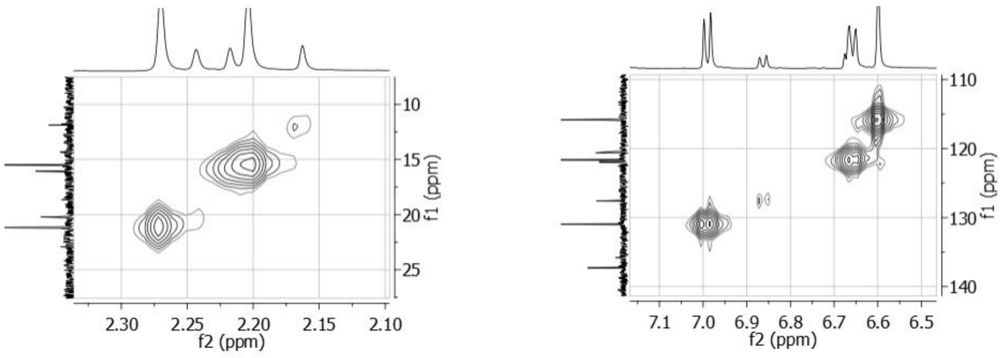

Figure S26. Plate picture of **6** MIC (minimum inhibitory concentration) against bacteria. Concentration ranged from 1000 to 125 μg/mL. P.C.: positive control; I. C.: inocule control; B. C. blank control.

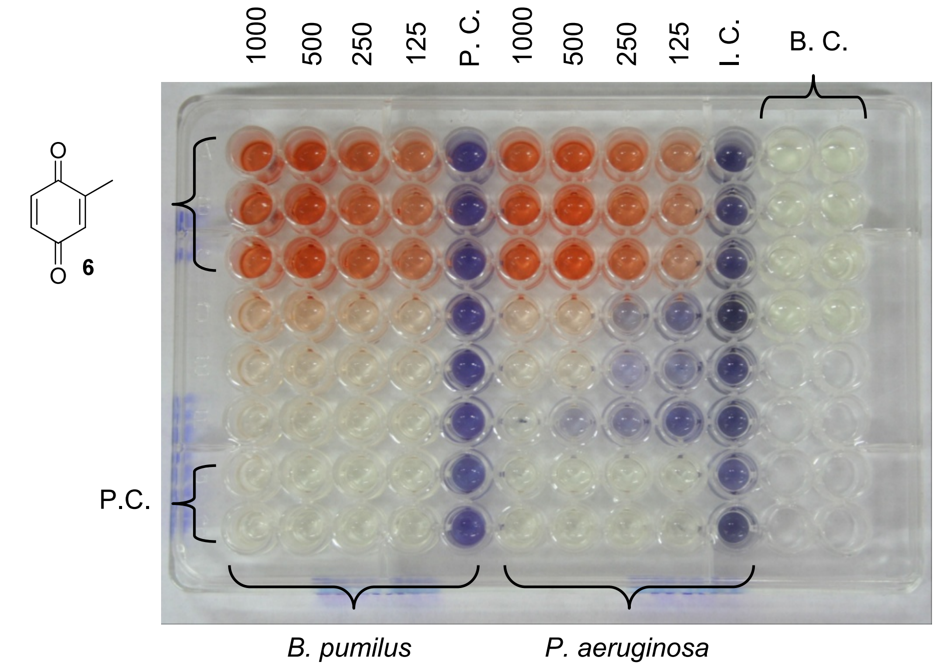

Figure S27. Plate picture of **8** and **18** MIC (minimum inhibitory concentration) against bacteria. Concentration ranged from 1000 to 125 μg/mL. P.C.: positive control; I. C.: inocule control; B. C. blank control.

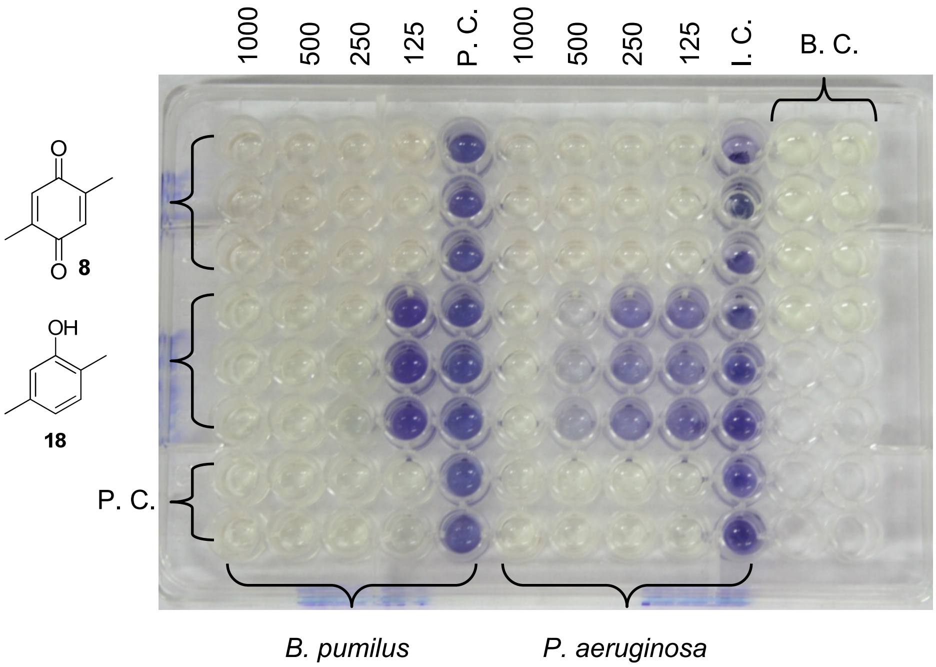

Figure S28. Plate picture of **6** MIC (minimum inhibitory concentration) against yeast. Concentration ranged from 500 to 82.5 μg/mL. P.C.: positive control; I. C.: inocule control; B. C. blank control.

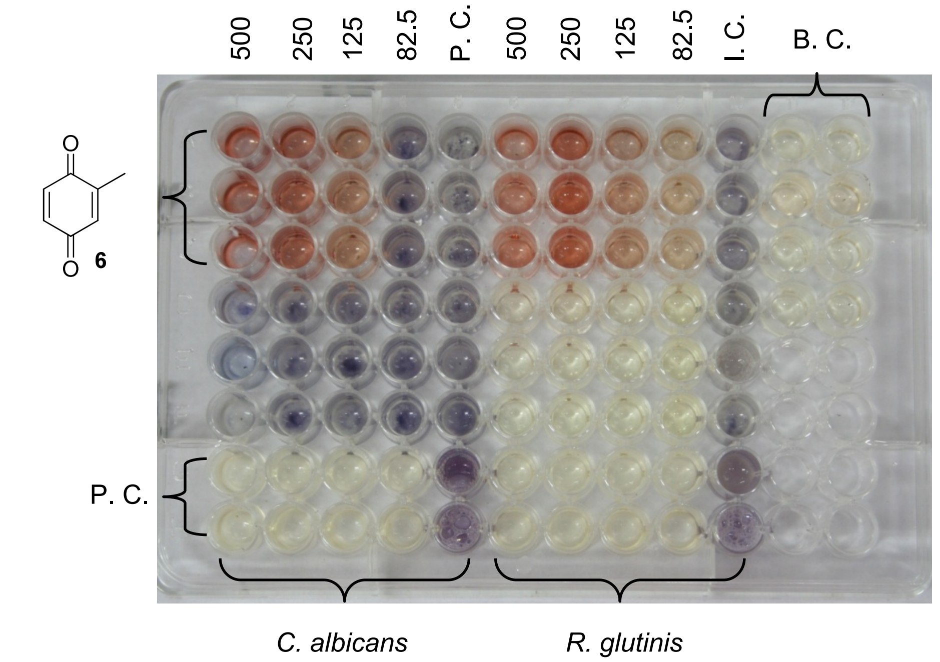

Figure S29. Plate picture of **8** and **18** MIC (minimum inhibitory concentration) against yeast. Concentration ranged from 500 to 82.5 μg/mL. P.C.:positive control; I. C.: inocule control; B. C. blank control.

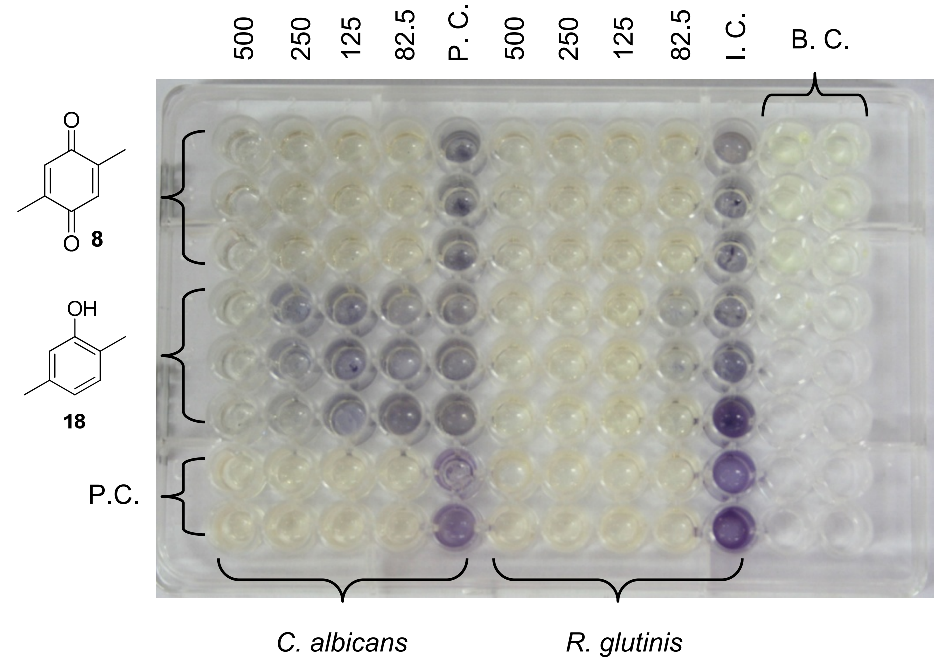

Supplement: Supplementary file 1 [file molecules-18-11429-s001.pdf]
